# Novel Galectin-3 Roles in Neurogenesis, Inflammation and Neurological Diseases

**DOI:** 10.3390/cells10113047

**Published:** 2021-11-05

**Authors:** Luana C. Soares, Osama Al-Dalahmah, James Hillis, Christopher C. Young, Isaiah Asbed, Masanori Sakaguchi, Eric O’Neill, Francis G. Szele

**Affiliations:** 1 Department of Physiology, Anatomy and Genetics, University of Oxford, Sherrington Building, South Parks Road, Oxford OX1 3QX, UK; luana.campossoares@dpag.ox.ac.uk (L.C.S.); isaiah.krikor@gmail.com (I.A.); 2 Department of Oncology, University of Oxford, Oxford OX1 3QX, UK; eric.oneill@oncology.ox.ac.uk; 3 Irving Medical Center, Columbia University, New York, NY 10032, USA; oa2298@cumc.columbia.edu; 4 Massachusets General Hospital, Harvard Medical School, 15 Parkman Street, Boston, MA 02114, USA; jamesmichaelhillis@gmail.com; 5 Department of Neurological Surgery, University of Washington, 325 Ninth Avenue, Seattle, WA 98104, USA; chris.uct@gmail.com; 6 International Institute for Integrative Sleep Medicine, University of Tsukuba, Tsukuba 305-8575, Japan; masanori.sakaguchi@gmail.com

**Keywords:** galectin, Galectin-3, neurogenesis, subventricular zone, stem cells, inflammation

## Abstract

Galectin-3 (Gal-3) is an evolutionarily conserved and multifunctional protein that drives inflammation in disease. Gal-3’s role in the central nervous system has been less studied than in the immune system. However, recent studies show it exacerbates Alzheimer’s disease and is upregulated in a large variety of brain injuries, while loss of Gal-3 function can diminish symptoms of neurodegenerative diseases such as Alzheimer’s. Several novel molecular pathways for Gal-3 were recently uncovered. It is a natural ligand for TREM2 (triggering receptor expressed on myeloid cells), TLR4 (Toll-like receptor 4), and IR (insulin receptor). Gal-3 regulates a number of pathways including stimulation of bone morphogenetic protein (BMP) signaling and modulating Wnt signalling in a context-dependent manner. Gal-3 typically acts in pathology but is now known to affect subventricular zone (SVZ) neurogenesis and gliogenesis in the healthy brain. Despite its myriad interactors, Gal-3 has surprisingly specific and important functions in regulating SVZ neurogenesis in disease. Gal-1, a similar lectin often co-expressed with Gal-3, also has profound effects on brain pathology and adult neurogenesis. Remarkably, Gal-3’s carbohydrate recognition domain bears structural similarity to the SARS-CoV-2 virus spike protein necessary for cell entry. Gal-3 can be targeted pharmacologically and is a valid target for several diseases involving brain inflammation. The wealth of molecular pathways now known further suggest its modulation could be therapeutically useful.

## 1. Introduction

**Figure 1:**
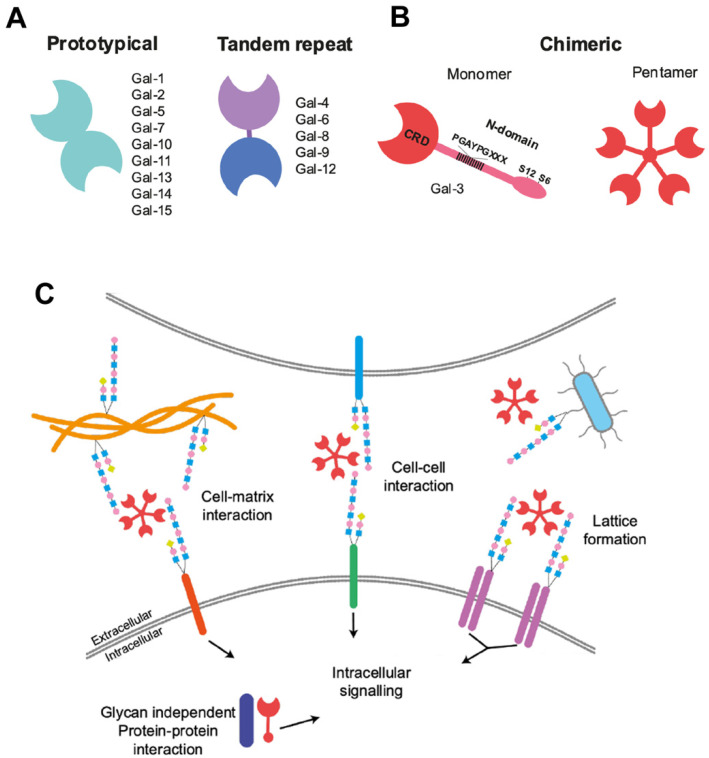
Schematics of Galectin-3 structure and function (**A**) Galectin subtypes and homology. Adapted from [4], (**B**) *N*-glycan binding partners of Gal-3. Adapted from [5], (**C**) Galectin-3 interactions. Adapted from [4], with permission.

Galectins are an evolutionarily conserved class of proteins being found in sponges, other invertebrates and mammals [1]. These lectins bind glycosylated proteins and lipids and can be located extracellularly, in the cell membrane and intracellularly. Galectin-3 (Gal-3) is unique amongst the 15 member galectin family (Figure 1A). Gal-3 is “chimeric” by virtue of a c-terminal carbohydrate recognition domain (CRD) and an *N*-terminal non-lectin domain that multimerizes the protein (Figure 1B). In contrast, galectin-1 (Gal-1), a prototypical galectin, has two CRDs and can be anti-inflammatory [2]. Gal-3 binds to *N*-glycans on receptors (Figure 1B), extracellular matrix proteins and pathogens and selectively mediates several key intracellular signaling pathways (Figure 1C). It thereby increases the release of several cytokines and has important functions in inflammation, the immune system and cancer. Gal-3 was previously termed “Mac-2” and was used as a putative marker for activated brain microglia and macrophages. More than just being a marker for them, Gal-3 is secreted by activated macrophages, and is a chemoattractant to them creating a feed-forward cycle [3].

Gal-3 acts as an arsonist at the early stage of inflammation but can act as a fireman at later stages. Gal-3 is upregulated in humans in many CNS diseases associated with inflammation including Alzheimer’s, stroke and hypoxia/ischemia [6,7]. It is also upregulated in rodent models including nerve axotomy, Alzheimer’s disease, multiple sclerosis, stroke and hypoxia/ischemia [8,9,10,11]. We and others find that Gal-3 loss-of-function results in specific histopathological outcomes depending on the disease and its severity. This specificity is likely explained by Gal-3 binding and modulation of divergent glycosylated molecules, including extracellular and intracellular proteins, suggesting it has many functions (Table S1). Gal-3 was shown to act as a natural paracrine ligand for the Toll-like receptor 4 (TLR4) and to thereby be responsible for inflammation in the lipopolysaccharide (LPS) model of bacterial infection [12]. A number of groups and companies have explored targeting Gal-3 for illnesses including heart disease and fibrosis and several phase II and III clinical trials are in progress. To our knowledge there are no clinical efforts to target Gal-3 in brain disease and inflammation, but given its disease-specific mechanisms of action we believe this a logical next step. It would be important to determine if the Gal-3 inhibitors available so far cross the blood–brain barrier (BBB). If they do, then testing their efficacy in models of brain diseases would be important. If the current inhibitors do not cross the BBB, a new class of Gal-3 inhibitors, capable of crossing the BBB, needs to be developed.

Gal-3 binds extracellular proteins including laminin [13,14], hensin [15], elastin [16], collagen IV [17], tenascin-C, and tenascin-R [18]. On the cell surface, its binding partners include receptors that mediate multiple signaling pathways (Table S1). Intriguingly, Gal-3 binding can be tissue specific. For example, Gal-3 binds EGFr in non-CNS cell-lines [19] and in solution [20], but we found that Gal-3 does not co-immunoprecipitate with EGFr in mouse SVZ tissue [7,21]. Similarly, while Gal-3 binds VEGFR2 in human umbilical vein endothelial cells [22], this was not the case in the mouse SVZ (Szele lab, unpublished). These discrepancies may be explained by the same protein displaying different tissue-specific glycosylation patterns, a scenario occurring for LRP1, the Prolow-density lipoprotein receptor-related protein 1 [23]. Inside the cell, Gal-3 binds to a different set of proteins. For example, Gal-3 complexes with Rb-CDK4-Cyclin D1 [24], K-Ras [25], Bcl2 [26], β-catenin/Tcf-4 [27,28] and components of the endosomal complex required for transport (ESCRT) [29,30]. For a more extensive list of Gal-3 binding partners, the reader is referred to excellent earlier reviews [31,32,33].

## 2. Galectin-3 Regulates Adult Subventricular Zone Cell Migration

Many more roles for Gal-3 have been uncovered in disease than in homeostasis. Not surprisingly therefore, Gal-3 expression is low in the healthy brain. However, robust Gal-3 expression in the subventricular zone (SVZ) neurogenic stem cell niche in healthy mice is a notable exception. This is of particular interest since SVZ microglia exhibit semi-activated morphologies, even in the absence of injury [34,35]. SVZ microglial morphology ranges from resting ramified to aggressive amoeboid and they express high levels of CD45, and divide more frequently than parenchymal microglia [34,35]. These findings gave clues that Gal-3 may have homeostatic functions in the SVZ niche in healthy brains, and these are described below.

The adult SVZ, which lines the lateral ventricles (LVs) and is the largest neurogenic stem cell niche in the mammalian brain, was originally described in detail by Joseph Altman in the 1960’s, using tritiated thymidine and classic histological methods [36,37]. The SVZ stem cell niche and neurogenesis can be influenced by growth factors, signaling molecules and transcription factors [38,39,40]. SVZ cell types can be distinguished with stage-specific markers, cell-cycle duration, morphological features and transcriptomics [41]. Thus, its cell composition, lineage relationships, molecular mechanisms and responses to disease are fairly well understood. The SVZ contains neural stem cells (NSCs) that proliferate and locally produce transit-amplifying cells (TAPs). TAPs then proliferate and eventually differentiate into neuroblasts, which migrate through the rostral migratory stream (RMS) into the olfactory bulbs (OB) where they mature into specific subtypes of OB interneurons. The adult-born OB neurons are implicated in multiple facets of odor detection, discrimination and memory [42]. Recently there have been a flurry of very useful single cell RNA sequencing (scRNAseq) papers that have indicated even more heterogeneity in the SVZ than previously expected [43,44,45,46,47]. These studies have also found microglia in the SVZ and showed that T cells infiltrate into the aging SVZ in humans [48] and contribute to decreasing neurogenesis in mice [49]. This is of interest since Gal-3 can regulate T cell proliferation, apoptosis and SVZ entry [50].

Neurons are also generated from stem cells in the subgranular zone (SGZ) of the hippocampal dentate gyrus (DG). The SGZ is of particular importance in humans, as it is implicated in memory and affective behaviors, and SGZ neurogenesis is decreased in Alzheimer’s disease (AD) [51,52]. The large majority of mammals that have been investigated exhibit adult neurogenesis. Human SVZ cells are also neurogenic in the first year of life with various groups finding they give rise to olfactory bulb, striatal or cortical neurons [53,54,55]. There is also evidence for human SGZ neurogenesis throughout life with estimates for significant lifetime replacement [52,53]. Whereas some studies cannot find human hippocampal neurogenesis [56], a well-controlled study from the Llorens-Martin lab recently confirmed adult human hippocampal neurogenesis in neurologically healthy individuals [52].

As mentioned above, Gal-3 is not immunohistochemically detectable in most of the brain, but we found it is expressed in the SVZ and the RMS in healthy mice [7,21]. Since Gal-3 is a classic marker of microglia, we expected strong expression in SVZ microglia as they are semi-activated [34]. However, Gal-3 was only minimally expressed by SVZ microglia. Instead it was found in neural cells, namely in ependymal cells (the highest expression), in glial fibrillary acidic protein positive (GFAP+) NSCs, and in some TAPs, but never in neuroblasts. This immunohistochemical expression pattern in the SVZ is strongly maintained in models including stroke [57], mild multiple sclerosis (MS) [58], and severe MS [50]. Transcriptomics analysis with singe cell RNAsequencing indicates a similar SVZ pattern as was found at the protein level, showing LGals3 mRNA is present in astrocyte-like NSCs, TAPs, but not in neuroblasts [59]. That study also found LGals3 transcripts in ependymal cells, smooth muscle cells, microglia and perivascular macrophages [59]. Interrogation of the Allen Brain Atlas shows positive Gal-3 in situ hybridization signals in the SVZ, further indicating Gal-3 is transcribed and translated in the niche rather than diffusing into it. The unique SVZ expression begged the question whether Gal-3 has a function in SVZ homeostasis. We found that loss of Gal-3 in knockout mice did not affect the number of BrdU+ label-retaining NSCs, nor the number of mitotic or apoptotic cells in the SVZ [21]. However, Dcx+ neuroblast chains became disrupted, 2-photon time-lapse microscopy showed reduced speed and straightness of neuroblast migration and overall rates of OB neurogenesis were reduced. These findings suggested that Gal-3 is necessary for maintaining SVZ neuroblast motility [21]. This effect was surprising since Gal-3 is not expressed by the neuroblasts themselves and suggested that Gal-3 was interacting with a cell surface protein. In parallel studies, we found that a subset of neuroblasts continue to express the EGFr and these EGFr+ neuroblasts were slower and less direct in their routes to the OB than EGFr-negative cells [60]. The similarity to the *Gal-3*^−/−^ results on neuroblast migration suggested Gal-3 affects EGFr function and indeed *Gal-3*^−/−^ SVZ cells had higher levels of EGFr phosphorylation [21].

## 3. Galectin-3 Functions in Gliogenesis and Gliomagenesis

### 3.1. Gal-3 Regulates Postnatal Gliogenesis

Since we showed Gal-3 expression and function in the adult SVZ niche, we looked for and found Gal-3 transcripts in the developing embryonic brain (Szele lab unpublished). Future studies could elucidate the role of Gal-3 in embryonic brain development as well as in maternal immune activation paradigms. We next detected Gal-3 in the P5 murine SVZ, in the same cells as adults-NSCs and astrocytes, ependymal cells, and some TAPs and few microglia, but not neuroblasts [7,21]. Supporting this, we showed Gal-3 is expressed in postnatal SVZ neurospheres which are composed of NSCs and progenitors [7,28]. The postnatal SVZ is a major source of forebrain glia [61] and Gal-3 roles in this process were important to study since it had been shown to affect glial biology later in life [62].

Gal-3 activates microglia and inflammation in human pathology and in models of disease such as stroke [63], Huntington’s disease [64], and multiple sclerosis (MS) [50]. However, it was unclear if increased Gal-3 activates microglia in the absence of injury. We therefore overexpressed Gal-3 in the healthy neonatal SVZ and showed that this did not affect microglia activation markers, numbers and morphology [7]. Thus, a model emerged suggesting Gal-3 induces microglial activation only when coupled with tissue damage, such as occurs in stroke, infection, or neurodegeneration. However, this did not rule out other homeostatic effects of Gal-3 in the postnatal brain. Therefore, we studied gliogenesis and neurogenesis in postnatal Gal-3 loss-of-function mice. Floxed Gal-3 conditional knockouts and Gal-3 knockdown reduced gliogenesis but not neurogenesis [7]. In contrast, Gal-3 overexpression in the SVZ increased astrocyte production and maturation in the striatum, while decreasing oligodendrocyte production. Gal-3 overexpression also reduced SVZ proliferation and increased cell-cycle exit [28]. Gal-3 regulates developmental signaling pathways and we asked if Wnt/β-catenin or bone morphogenic protein (BMP) signaling were involved in Gal-3’s postnatal function.

Firstly, Wnt/β-catenin signaling regulates numerous functions in the SVZ and we showed Gal-3 binds β-catenin in SVZ cells [28]. Wnt regulates NSC maintenance [65], symmetric division of NSCs [66] and TAPs [67], as well as oligodendrocytic fate and neuronal differentiation [68,69], suggesting Gal-3/β-catenin binding could have important functions. Inflammation and Wnt signaling blockade mediates age-related NSC quiescence [43] suggesting Gal-3 may play a role in the aging SVZ. We showed that Gal-3 knockdown in the SVZ increased Wnt signaling, whereas overexpression reduced it [28]. Gal-3 may regulate Wnt signaling via binding to β-catenin, sequestering it and inhibiting transcriptional regulation. Whereas Gal-3 reduced Wnt signaling in the SVZ it increases it in pancreatic, breast, colon and tongue cancers [27,70,71,72], as well as in glioblastoma cell-lines (Al-Dalahmah, O–unpublished). The contrast between cancer cell-lines and benign NSCs is of great interest and suggests that tumorigenic transformation alters how Gal-3 regulates Wnt signaling, which remains an open question (please see Section 3.2).

Secondly, we also showed that Gal-3 regulates BMP signaling in the SVZ and binds to the BMPR1A [7]. BMP 2, 4, 6, and 7 ligands and their receptor serine threonine kinases BMPR1A, BMPR1B, and BMPR2, as well as the BMP inhibitor Noggin, are all expressed in the postnatal SVZ niche [73,74]. In the adult SVZ, baseline BMP signaling is required for neurogenesis [73], but increasing BMP levels reduces proliferation and neurogenesis [74,75,76]. BMP signaling also regulates SVZ fate choices by promoting astrocyte generation [77,78,79] and suppressing oligodendrogenesis [73,80,81]. In our study, Gal-3 increased BMP signaling as indicated by increased Smad1/5/8 phosphorylation [7]. Conditional loss of the BMPR1A combined with Gal-3 over expression blocked the increased astrocyte to oligodendrocyte ratio. Interestingly, Gal-3 knockdown did not decrease BMP signaling, suggesting that baseline BMP signaling is maintained via multiple pathways. Together, our results show that Gal-3 coordinates BMP and Wnt signaling in the postnatal SVZ NSCs independent of inflammation, and is a molecular switch for astrocyte genesis in the SVZ.

### 3.2. Gal-3 a Tumorigenesis Marker with Functional Relevance in Gliomagenesis

Cancer is multifactorial with molecular characteristics supporting tumor proliferation and infiltration [82]. Gal-3’s pro-inflammatory role and upregulation of several pro-tumorigenic pathways is linked with cancer aggressiveness. Inflammation, via cytokine secretion and immune cell recruitment, is an attempt to control tumor growth but can instead stimulate tumorigenesis and genomic instability [83,84,85,86,87,88]. Cytokines can activate pathways such as Pi3K/AKT and the Jak/STAT in dedifferentiating tumors and augment cancer stem cells [89,90]. Gal-3 can act at different tumor stages, on multiple cell types and subcellular localizations, a phenomenon not yet completely understood in the context of gliomagenesis. For example, endothelial cell Gal-3 expression is high in low grade glioma but almost absent in glioblastoma, [91,92,93]. In contrast Gal-3 is highly expressed in the parenchyma, in the hypercellular regions surrounding necrosis, supporting its importance for migration or survival of those cells [91]. Gal-3 also modulates the glioma microenvironment via interaction with the glycoprotein chitinase-3-like 1 (CHI3L1), increasing tumor immunosuppression and promoting macrophage M2-polarization [94]. Interestingly, this interaction can be inhibited by Gal-3 binding protein [94].

Gal-3 also has pro-survival roles in tumorigenesis both intra- and extra-cellularly. Gal-3 shares sequence similarity with the NWGR motif of Bcl-2, an antiapoptotic protein [26,95]. Gal-3 also directly binds to Bcl-2 and to other apoptotic proteins involved in apoptosis such as CD95, Nucling and synexin [96,97,98]. Crucially, Gal-3 promotes resistance to anoikis, adhesion-loss induced apoptosis, contributing to tumor dissemination [99,100]. Gal-3 also influences tumor spread through its interaction with integrins, triggering anchorage-independent signaling, facilitating stemness and metastasis [101,102,103].

The uniquely inflammatory status of the SVZ may predispose cancer development via pro-inflammatory regulators such as Gal-3 [35]. Gliomas contacting the SVZ are more aggressive, likely to spread and have a worse prognosis than those not in contact with the stem cell niche [104,105,106,107,108,109,110,111,112,113,114,115]. Gal-3 regulates gliomagenic developmental pathways in the SVZ, enhancing their stem cell characteristics and tumor aggressivity and Gal-3 is a component of the mesenchymal glioblastoma gene signature [116]. Seguin and colleagues have recently shown that Gal-3 regulates micropinocytosis in mesenchymal glioblastoma stem cells, via interaction with Ras related protein 10 (RAB10) and β1 integrin [117].

Cancer-secreted Gal-3 activates Notch signaling impairing differentiation [118,119]. As mentioned, Gal-3 can bind to *N*-glycan residues of tyrosine/kinase receptors EGFR and BMPr1α preventing endocytosis of the former, which ultimately results in upregulation of progenitor genes such as Sox2 [7,19,120]. Notch and EGFR signaling are activated in gliomas contributing to glioma stem cell maintenance [121,122,123,124]. Gal-3 secreted by cancer cells binds to the Notch receptor Jagged-1 and thereby activates angiogenesis [125]. As described above, Gal-3 activates BMP signaling, which controls glioma stem cell quiescence [126,127].

We described above our study showing that Gal-3 binds β-catenin and downregulates Wnt signaling in postnatal SVZ gliogenesis [28]. Wnt pathways are implicated in glioma malignancy and stemness and may be a therapeutic target [128]. Since Gal-3 in the SVZ modulates Wnt signaling opposite to how it is regulated in cancer, SVZ malignant transformation could require a Gal-3 functional switch. In breast cancer, Gal-3 can activate Wnt signaling by mediating β-catenin nuclear localization via direct β-catenin Gal-3 interactions and enhancing Wnt target gene transcription [27,73]. Gal-3 can also indirectly activate Wnt signaling via Akt and GSK3β downregulation in colon [73], pancreatic [72] and tongue cancers [72]. Additionally, Gal-3 can regulate the β-catenin destruction complex as it contains a GSK3β phosphorylation motif and associates with axin [129].

**Figure 2:**
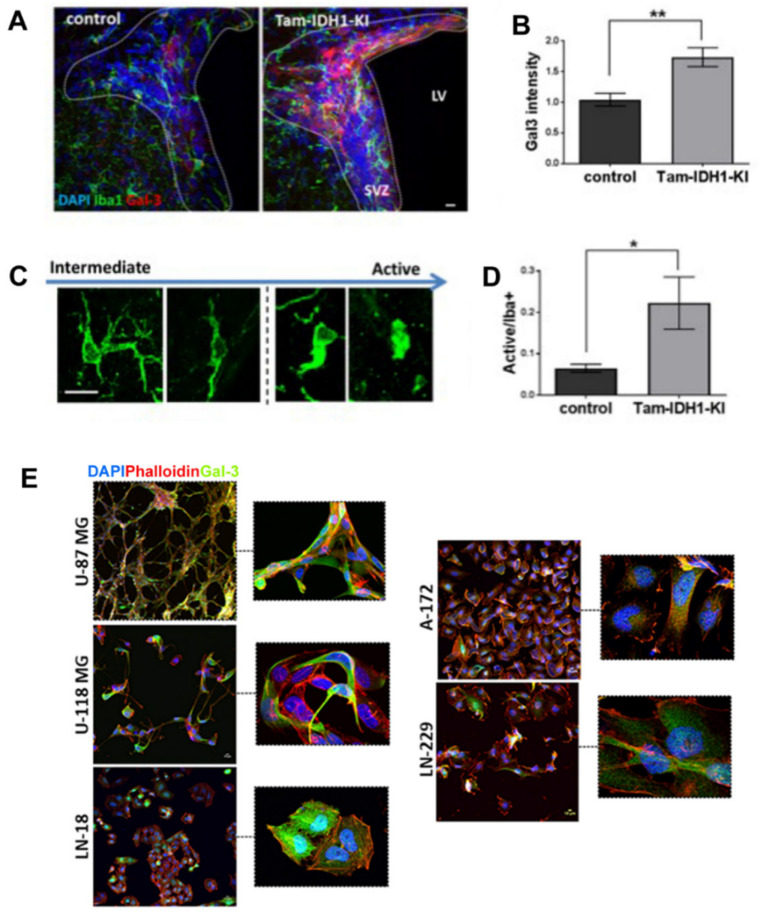
Galectin-3 expression and microglia in an SVZ cancer model and in cancer cells. (**A**) Gal-3 expression (red) and microglial Iba1 expression (green) are increased in the SVZ of the IDH1^R132H^ model of gliomagenesis as described in Bardella et al., 2016. Scale bar is 5 microns. (**B**) Quantification of A showing significantly increased Gal-3 in the glioma model SVZ. (**C**) Typical Iba1+ microglial morphologies in the SVZ ranging from intermediate to activated. (**D**) Quantification of C showing that in the IDH1 model there are significantly more active microglia in the SVZ. (**E**) Gal-3 expression (green) is found in five glioblastoma cell lines. Phalloidin (red) used to detect actin. ((**A**–**E**): Szele, Tomlinson, Bardella and O’Neil labs, unpublished data). * *p* < 0.05, ** *p* < 0.01.

To model early SVZ gliomagenesis, we generated a mouse with conditional IDH1^R132H^ expression in the niche. These IDH1^R132H^ knock-in mice exhibited heightened SVZ proliferation, stem cell expansion and infiltration into adjacent tissue [130]. Gal-3 SVZ expression and microglial activation are heightened in these mice (Figure 2A–D). The enzyme Mgat5 (beta1,6 *N*-acetylglucosaminyltransferase V) adds branched sugars to proteins and galectin binding is proportional to the number of branches [131]. Tumor microenvironments frequently alter glycosylation through abnormal Mgat5 function, which can then alter Gal-3 binding and function [132]. Mgat5 and branched *N*-glycans are related to early gliomagenesis, regulating proliferation and invasion [133,134,135]. These data suggest further Mgat5-mediated roles for Gal-3 in glioma formation and invasion. Gal-3’s actions in promoting brain tumorigenesis and its expression in multiple glioblastoma cell lines (Figure 2E) suggest it could be a good therapeutic target. Interestingly, Gal-3 conferred resistance to traditional treatment with chemotherapy and radiotherapy in glioblastoma [136]. Several inhibitors of Gal-3 have been described and some are in clinical trials for cancer [137,138].

## 4. Galectin-3 Actions in Multiple Sclerosis and Stroke

### 4.1. MS, Gal-3 and the SVZ

Multiple sclerosis (MS) is an autoimmune condition that targets the myelin sheath surrounding neuronal axons [139]. The pathophysiologic process, demyelination, is modeled in animals using both immunogenic and toxic approaches [140]. As expected given the inflammatory nature of demyelination, Gal-3 has an established role in MS. Since, the SVZ generates glia postnatally [61] and Gal-3 regulates SVZ oligodendrogenesis during early postnatal development, it follows that it may do so in regeneration. Interestingly, it has been suggested that SVZ-derived oligodendrocytes may be superior at re-myelination than those derived from oligodendrocyte precursor cells (OPC’s) [141]. Human MS activates the SVZ and its oligodendrocyte production during adult demyelination [142,143,144].

### 4.2. Mgat5-Mediated Sugar Branching Affects MS

SNPs (single nucleotide polymorphisms) in Mgat5 are linked to age of onset and severity in MS [145]. In the mouse MS models, EAE (experimental allergic encephalomyelitis) and TMEV (Theiler’s murine encephalomyelitis virus) susceptible strains exhibit 20-fold less GlcNAc (*N*-acetyl glucosamine) branching. Mgat5 knockouts in these strains exhibit spontaneous demyelination in 80% of mice and more severe EAE [146].

### 4.3. Gal-3 Expression following Demyelination in Humans

**Figure 3:**
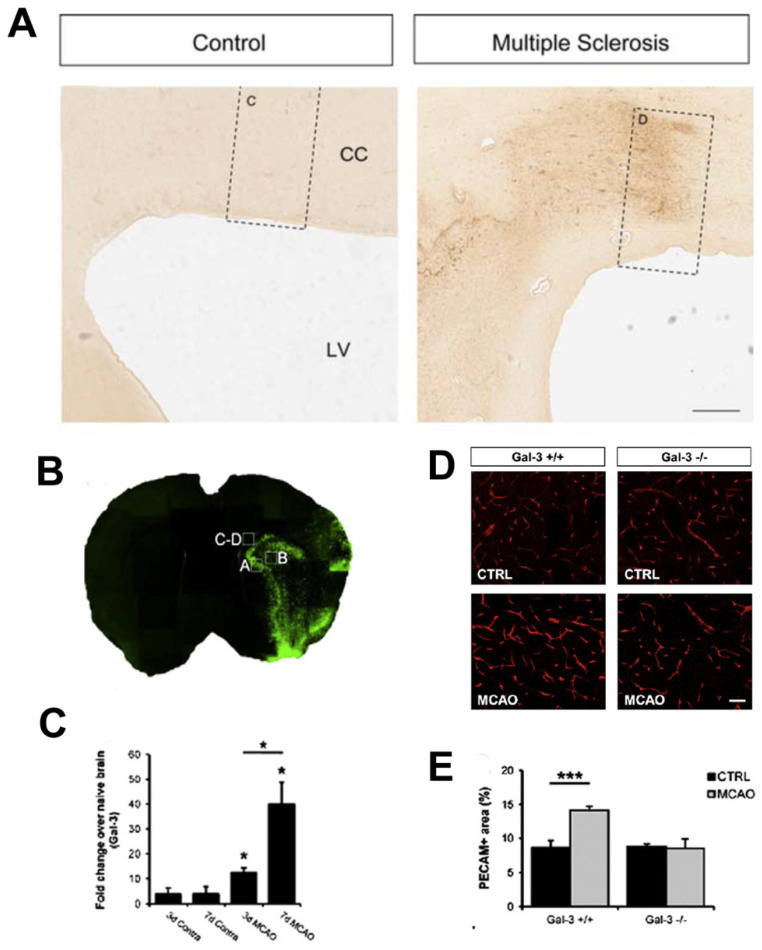
Galectin-3 is upregulated in multiple sclerosis and controls angiogenesis after stroke. (**A**) Gal-3 immunohistochemistry (brown) in human brain sections shows that compared to a healthy control, Gal-3 is increased near the lateral ventricle (LV) in an MS patient. (Adapted from [50]. (**B**) Gal-3 immunofluorescence (green) is increased in the striatum and cerebral cortex of a mouse middle cerebral artery occlusion (MCAO) stroke model. (**C**) Quantification of B showing significantly increased Gal-3 at days 3 and 7 post-stroke. (**D**) Platelet endothelial cell adhesion molecule (PECAM) blood vessel immunofluorescence (red) in the striatum of WT and *Gal-3*^−/−^ mice, with or without MCAO. (**E**) Quantification of D showing increased blood vessels after MCAO in controls but not in *Gal-3*^−/−^ mice. ((**B**–**E**) Adapted from [10]), with permission. * *p* < 0.05, *** *p* < 0.001.

Human post-mortem studies in MS find increased Gal-3 mRNA and protein expression (Figure 3A) [50,147,148]. Microglia, macrophages and hypertrophic astrocytes in MS lesions demonstrate Gal-3 expression [147]. Gal-3 is increased in active rather than chronic inactive MS plaques suggesting it actively modulates disease pathology [147]. Gal-3 increases more in primary progressive MS, where MS symptoms steadily worsen from onset, compared to secondary progressive MS, where MS symptoms initially relapse and remit before steadily worsening [148]. There is a particular prominence of Gal-3 expression in the periventricular white matter both within and outside of MS lesions [50]. This is important as MS lesions known as Dawson’s fingers emanate from the lateral ventricles into surrounding regions [149].

### 4.4. Animal Models of MS and Gal-3

Similar to human MS patients, animal demyelination EAE, cuprizone and TMEV models display increased Gal-3 levels [50,150]. Inflammation and Gal-3 increases are especially prominent around the lateral ventricles and SVZ in TMEV [50,151]. In studies in which we used the cuprizone toxin model, Gal-3 expression increased near the SVZ in the white-matter of the corpus callosum [58]. However, after cuprizone treatment Gal-3 expression decreased in the SVZ, a rare occurrence of decreased Gal-3 expression in a disease model. Similar to humans, Gal-3 appears to be localized to parenchymal macrophages, microglia and astrocytes in animal models of the disease, but in the SVZ it is mostly expressed by NSCs, TAPS and ependymal cells [50,58,150,152].

### 4.5. Gal-3 Microglial, Inflammation and Molecular Mechanisms in Demyelination

Gal-3 recruits and activates microglia providing a key role in demyelination. Both the EAE and TMEV models have greater microglial presence in Gal-3 wildtype (*Gal-3^+/+^*) compared to knockout (*Gal-3*^−/−^) mice [50,153]. In the EAE model, the increased microglial presence is associated with increased clinical severity. The cuprizone model has conflicting literature, although it is important to consider the differences in the *Gal-3*^−/−^ mice models used in these studies: not only was the background strain different but, in Hoyos et al. (2014) Gal-3 had an interruption in the CRD by insertion of the neomycin resistance gene at the intro 4-exon 5 junction, while Hillis et al. (2016) eliminated exons 2, 3 and 4 which encode part of both protein domains. Hoyos et al. (2014) report that *Gal-3^+/+^* mice have fewer, yet more activated, microglia than *Gal-3*^−/−^ mice after cuprizone treatment, whereas Hillis et al. (2016) find no difference in the number of hematopoietic cells [58,152]. For microglial activation in the cuprizone model, Gal-3 increases expression of the phagocytic receptor TREM-2b [152]. It has also been proposed that Gal-3 binds to K-Ras-GTP to activate PI3K and phagocytosis [154]. Finally, Gal-3 may be required for matrix metalloproteinase-3 activity, which in turn activates microglia [155,156].

Gal-3 is upstream and required for several pro-inflammatory molecules in MS. Microglia are recruited to the pro-inflammatory milieu of EAE, which Jiang et al. (2009) studied extensively. *Gal-3*^−/−^ lymph nodes produced less interleukin 17 (IL-17), interferon gamma (IFN-γ) and IL-6 in the presence of EAE-inducing myelin oligodendrocyte glycoprotein antigen [157]. In the CNS, *Gal-3*^−/−^ mice but not WT mice had detectable IL-17, IFN-γ, tumor necrosis factor alpha (TNF-α), and inducible nitric oxide synthase (NOS) transcripts. IL-17 promotes blood–brain barrier disruption allowing T(H)17 cell infiltration in MS pathogenesis [139,158]. Gal-3 autoantibodies have separately been suggested to disrupt the blood–brain barrier by binding to Gal-3 on brain microvascular endothelial cells and increasing expression of intercellular adhesion molecule-1 and phospho-nuclear factor-kappa B p65 [159]. In other studies we found greater expression of the chemokines CCL2, CCL5, CCL8 and CXCL10 in the SVZ after TMEV treatment in *Gal-3^+/+^* compared to *Gal-3*^−/−^ mice [50]. We also found that many other chemokine and cytokine expression profiles were decreased in *Gal-3*^−/−^ mice after TMEV (Table S2) [50]. More recent work upholds the general principle that Gal-3 is pro-inflammatory in the CNS by showing that absence of Gal-3 reduces neuroinflammation in acute peripheral inflammation mouse models [157].

### 4.6. Gal-3 Affects Oligodendrocyte Differentiation

As previously discussed, Gal-3 aids oligodendrocyte differentiation in normal postnatal mice. Separate from its immunologic role in diseases, Gal-3 promotes oligodendrocyte differentiation in demyelination. Gal-3 added to oligodendrocyte cell lines increased differentiation and further increased Gal-3 expression during differentiation [160]. CSF from primary progressive MS patients added to rat oligodendrocyte precursor cells increased Gal-3 expression and increased the number of primary branches from the soma suggesting differentiation [148]. Conversely, in the absence of Gal-3, fewer neurosphere progenitor cells committed to oligodendrocyte fate, and in vivo experiments show decreased myelin integrity in the absence of Gal-3 [160]. *Gal-3^+/+^* but not *Gal-3*^−/−^ mice demonstrated spontaneous remyelination five weeks after cuprizone [152]. Interestingly, *Gal-3*^−/−^ mice demonstrated increased oligodendrocyte precursor cells both at rest and during cuprizone treatment, and had fewer multipolar processes suggesting arrested differentiation [152]. Thus, in aggregate the data suggest Gal-3 is necessary for oligodendrocyte differentiation.

### 4.7. Galectin-3 Functions in Adult Stroke

Stroke leads to a cascade of inflammatory changes and is a leading cause of mortality and disability. Currently, beyond the first few hours where medical thrombolysis and mechanical thrombectomy have yielded impressive results, no treatment is available once the ischemic injury has become established and therefore regenerative approaches are being examined [161]. Gal-3 is garnering clinical interest and serum Gal-3 levels may be useful as a predictor of stroke severity and clinical outcome [162,163]. Given Gal-3’s neuroinflammation roles, it is well-positioned to influence tissue remodeling following ischemic injury. Continued understanding of Gal-3 and its role in post-stroke angiogenesis, neurogenesis and neuroinflammation could contribute to the development of future diagnostic and therapeutic strategies.

To better understand the effect of Gal-3 in stroke, we performed middle cerebral artery occlusion (MCAO) stroke in *Gal-3*^−/−^ knockout mice and compared them to *Gal-3^+/+^* controls. Gal-3 was increased in the area of injury (Figure 3B,C) and deletion of Gal-3 selectively inhibited the stroke-induced increases in endothelial cell proliferation and density in the ischemic penumbra (Figure 3D,E) [10]. Vascular endothelial growth factor (VEGF) and its tyrosine kinase receptors are key regulators of post-stroke endothelial proliferation [164,165]. In *Gal-3*^−/−^ mice, the inhibition of post-stroke angiogenesis was associated with attenuation of the expected upregulation of VEGF, and may be a mechanism for the inhibited endothelial proliferation in *Gal-3*^−/−^ mice after stroke. In contrast to other studies, the reduction in post-stroke angiogenesis in *Gal-3*^−/−^ mice impacted neither stroke size nor functional outcomes [10]. Strategies which increase post-stroke endothelial proliferation appear to reduce stroke size and improve functional outcome, findings which have prompted clinical trials designed to increase angiogenesis. Loss of Gal-3 affected neither inflammation nor proliferation nor neurogenesis in the SVZ. SVZ neuroblasts are diverted from their normal migratory pathway and migrate to the site of ischemic injury. To our surprise, loss of Gal-3 did not affect post-stroke migration of neuroblasts towards the ischemic penumbra. Cytoarchitectural changes such as astrogliosis, endothelial proliferation and loss of ependymal planar cell polarity in the SVZ following ischemic stroke [57] were not affected by *Gal-3*^−/−^ [10]. These results suggest that Gal-3 function in the SVZ can diverge substantially from non-neurogenic parenchymal brain regions. Recent work has continued to indicate that Gal-3 is correlated with and impacts stroke outcomes. Levels of Gal-3 in the serum of patients is associated with severity and progression of ischemic stroke [166]. Treatment with melatonin after ischemic stroke is neuroprotective, reduces levels of Gal-3 and ameliorates hyperactivity and anxiety in rats [167]. However, a recent study showed that treatment with Gal-3 is protective to stroke (MCAO in rats), preventing apoptosis and neurodegeneration [168]. Gal-3 promoted activation of pro-survival pathways such as Akt; and downregulation of pro-apoptotic proteins such as ERK and Caspase-3 [168].

Because of the multiple Gal-3 binding partners and signaling effects, we sought to determine if loss of Gal-3 changes gene expression. We examined striatal protein expression in wild type (WT) and *Gal-3*^−/−^ mice in the presence or absence of MCAO (Table S3) [10]. Remarkably, Gal-3 loss induced a greater than 10-fold decrease in prolactin expression. *Gal-3*^−/−^ mice also had broad increases in insulin-like growth factor binding proteins IGFBP-1,2,3,9,10, suggesting it regulates insulin signaling. Interestingly, MCAO-induced increases in IGFBP expression were further increased in *Gal-3*^−/−^ compared to WT mice. The same scenario was seen in the expression of thrombospondin-2 and of ADAMTS1 (a disintegrin and metalloproteinase with thrombospondin motifs), suggesting Gal-3 normally limits expression of certain genes involved in angiogenesis.

### 4.8. Gal-3 Functions in Neonatal Hypoxia Ischemia

Gal-3 gene expression is similarly upregulated in a murine model of neonatal hypoxic-ischemic (H/I) injury [169] Unlike our study where deletion of Gal-3 did not affect stroke size and functional outcome, loss of Gal-3 was neuroprotective resulting in reduced ischemic tissue volume [169]. Unexpectedly, this was associated with increased microglial activation and insulin growth factor 1 (IGF-1) expression in *Gal-3*^−/−^ mice. In contrast, targeted ablation of Gal-3+ microglia/macrophages in MCAO was associated with decreased IGF-1 levels but increased apoptosis and stroke size [170]. The same group also demonstrated that deletion of Gal-3 resulted in larger stroke size following MCAO, due to impaired microglial activation and IGF-1 production [170]. In another study in rats, treatment with anti-Gal-3 antibodies attenuated post-stroke endothelial and neural progenitor proliferation in the ischemic striatum and SVZ, respectively [171]. There was no change in stroke size and functional outcome was not reported [171]. In other work, Gal-3 reduced the microglial pro-inflammatory response to LPS and TNFα in vitro [172]. Gal-3 also restored IGF-1 levels after LPS treatment but the same results were not observed in vivo in a model of neonatal hypoxic-ischemia [172].

## 5. Galectin-3 Can Exacerbate Alzheimer’s Disease and Diabetes

### 5.1. Gal-3 Elicits Alzheimer’s Pathology and Symptoms

Amongst molecules implicated in Alzheimer’s disease (AD), Gal-3 has recently emerged as one of the most promising, mechanistically and therapeutically [6,173]. Gal-3 concentrations are increased in the brains, CSF and plasma of humans with AD [174,175,176] and Gal-3 brain injections increase insoluble Aβ levels and toxicity in animals [11,177]. Endogenous Gal-3 is most highly expressed in Aβ plaques [11]. As well, Gal-3 expression increased with age, paralleling Aβ oligomerization [177]. Importantly, SNPs in the Gal-3 gene (LGALS3) are associated with increased risk for AD [11]. Loss of Gal-3 in the severe 5XFAD mouse model of AD decreased microglial Toll-like signaling as well as the AD signature TREM2/DAP12 signaling [11]. Reduced Gal-3 in hemizygote APP/PS1 mice decreased inflammation and enhanced cognition compared to controls [177]. Loss of Gal-3 also ameliorated hippocampus-dependent cognition, suggesting that increased Gal-3 in AD could exacerbate this symptom [11,177]. Another group showed that Gal-3 binding protein (GAL3BP) inhibits β-secretase processing of amyloid precursor protein and thereby reduces Aβ [178].

AD frequently commences in the hippocampus and hippocampal neurogenesis decreases in AD [52]. Stimulating adult hippocampal neurogenesis in rodents improves memory and pattern separation whereas reduced hippocampal neurogenesis decreases memory functions [179,180]. Interestingly, rats undergoing contextual fear conditioning or spatial memory training, which are also adult neurogenesis-dependent behaviors, exhibited reduced hippocampal Gal-3 expression [181]. Reducing Gal-3 increased memory in these tests, whereas overexpression diminished memory, suggesting that Gal-3 normally limits these functions [181]. Whilst all this is intriguing, a major gap in our knowledge is whether Gal-3 directly affects hippocampal neurogenesis, as does Gal-1 (described below).

### 5.2. The Links between AD, Diabetes, Gal-3 and Insulin

A remarkable study showed that in obesity, adipose tissue macrophages secrete Gal-3, which is a chemoattractant to macrophages which then secrete even more Gal-3, in a feed-forward cycle [3]. Gal-3 then binds to the insulin receptor and blocks it, causing insulin resistance [3]. In addition to the insulin receptor, Gal-3 binds to the insulin-like growth factor 1 receptor (IGF1R) [63]. Brain insulin modulates cognition yet how it does so is unclear [182]. These studies are of great interest since the Levison group and others have shown that insulin, IGF-1 and IGF-2 influence various aspects of adult neurogenesis [183]. Whether increased or decreased levels of Gal-3 affect insulin and IGF function in adult SVZ or SGZ neurogenesis is unknown. Type 2 diabetes (T2D) models were recently shown to have decreased hippocampal neurogenesis in both db/db mice (obesity dependent) and IGFr mutant mice (obesity independent) [184]. Hippocampal neurogenesis decreases in human T2D and Gal-3’s role in diabetes may impact neurogenesis [3,185,186]. AD and T2D have significant co-morbidity [187] and given the impact of Gal-3 in AD, it will be important to compare its role in the two diseases. Cerebral microbleeds are common on diabetes melitus and Gal-3 expressing macrophages were associated with abnormal elimination of microvessels at sites of microbleed [188]. Depletion of these macrophages resulted in microvessel repair [188].

## 6. Galectin-3 Relevance in COVID-19 Brain Pathology

### 6.1. Galectins Mediate Viral Infection

A survey of the literature indicates that galectin expression is increased by many viruses via transcriptional regulation or otherwise [189,190]. Galectins as a family have broad roles in regulating viral infections [189,191]. They can act directly on viruses by binding glycosylated viral envelope (env) proteins [190]. Viral glycoproteins can profoundly affect virulence; a single amino acid mutation in the Zika virus env protein caused the 2015 pandemic [192]. Gal-1 is known to bind to the influenza virus and also to decrease flu symptoms [193]. Gal-9, another galectin expressed in the SVZ, binds the human immunodeficiency virus (HIV) virus and HIV increases its expression [190]. The SARS-CoV-2 virus is glycosylated with *N*- and *O*-linked glycan residues located on the receptor binding domain [194,195,196]. The ACE2 receptor is also glycosylated [197] and it is likely that Gal-3 binds to the virus and its receptor. Interestingly, GAL3BP has been shown to bind directly with SARS-CoV-2 and in cell culture models of infection, increasing GAL3BP levels blocked both viral SARS CoV-2 pseudoparticle cell entry and induced cell fusion [198].

### 6.2. Targeting Gal-3 in COVID-19

Two of the greatest risk factors for COVID-19 mortality are obesity and old age. Interestingly, Gal-3 expression is upregulated in both, suggesting it may contribute to the increased inflammation seen in obesity and in old age and that blocking Gal-3 may be a viable therapeutic target [3,11]. Gal-3 inhibitors are being developed for a number of diseases including fibrosis, heart disease and cancer [199,200,201,202,203]. An intriguing suggestion is that they be repurposed for blocking the SARS-CoV-2 virus [204]. This is a logical choice based on Gal-3’s role in inflammation and pathogen response. As mentioned above, Gal-3 is generally pro-inflammatory in the CNS and increases expression of many inflammatory cytokines, for example IL-6 and TNF-α expression via NFKβ [205]. Gal-3 also has well-known roles in infection and pathogen pattern recognition [206,207,208]. Another link is that the Gal-3 CRD shares structural features with coronavirus spike proteins in general [209,210]. The SARS-CoV-2 spike glycoprotein specifically shows remarkable similarity to the Gal-3 CRD. We agree with Caniglia, Velpula and colleagues that it is important to test the ability of these compounds to modulate COVID-19 and also to better understand Gal-3’s role in infection and prognosis of the disease [204].

### 6.3. Does Gal-3 Block Pathogen Entry through the SVZ?

**Figure 4:**
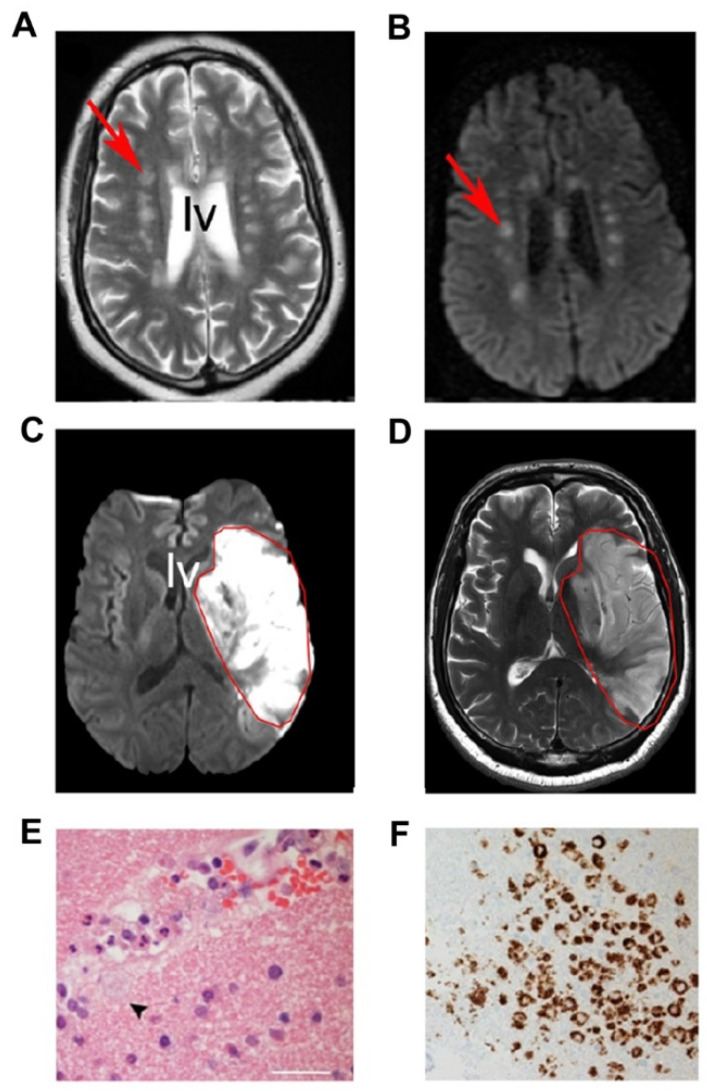
CNS pathology in COVID-19 victims. (**A**,**B**) MRI showing small foci of injuries (arrows) near the lateral ventricle (LV) and SVZ. (**C**,**D**) Large lesion (outlined in red) near the lateral ventricles. (**E**) A small blood vessel surrounded by immune cells that invaded the brain. Note macrophage extending into brain (small arrowhead). (**F**) CD68 immunohistochemistry showing macrophages around small vessels. (Adapted from Paterson et al., 2020), with permission.

An intriguing question is whether Gal-3 regulates infiltration of pathogens into the SVZ and the brain. SARS-CoV-2 is glycosylated and Gal-3 may intercept it in a proposed network of molecules. A detailed neurological study of CNS pathology reveals that in many cases of COVID-19, encephalopathy is adjacent to or directly impinges on the SVZ (Figure 4A–D) [211]. The SVZ lines the lateral ventricles and along with ependymal cells comprises the cerebrospinal fluid (CSF) brain barrier. However, the barrier is not perfect as SVZ NSC primary cilia extend amongst ependymal cells and contact the CSF in the lateral ventricles. Additionally, we found that loss of Gal-3 causes disruption of ependymal cell motile cilia [21]. We are not aware if increased Gal-3 also causes ciliary problems but if it does, virus could pool in the lateral ventricles. After MCAO stroke, ependymal planar cell polarity was disrupted and we had functional evidence of ciliary dysfunction [57]. Another scenario is that the virus could infect SVZ neuroblasts that would then spread the virus through the brain, since these progenitors frequently move out of the niche and into lesioned areas. The SARS-CoV-2 virus likely has tropism for sialic acid residues [212], and SVZ neuroblasts express polysialylated neural cell adhesion molecule (PSA-NCAM) [213]. In a remarkable instance of viral tropism for the SVZ, we found that the TMEV viral model of MS targets it selectively [50,151]. It is thus important to consider the links between viral entry into the brain via the CSF-brain barrier of lateral ventricles and the expression and function of Gal-3. Even if SARS-CoV-2 does not enter the brain via the lateral ventricles, it likely does through blood vessels disrupted by the virus (Figure 4E). These are frequently surrounded by reactive microglia (Figure 4F) which are likely regulated by Gal-3.

## 7. Galectin-1 Modulates Neurogenesis in the Healthy and Injured Brain

Together with Gal-3, Gal-1 is one of the most studied galectins in the brain. It is also commonly associated with pathological processes such as neuroinflammation. Increasing new evidence supports that Gal-1 and Gal-3 are not redundant molecules, even when expressed in similar contexts, but rather they have defined and specific actions. Therefore, it is relevant to consider the role of Gal-1 in the healthy and diseased brain. A more detailed comparison of Gal-1 and Gal-3 in neuroinflammation can be found in [214].

Amongst the 15 galectins, Gal-3 is chimeric and therefore in a class by itself, whereas Gal-1, the first galectin to be discovered, belongs to the “prototypcal galectins”, a subfamily including Gal 1, 2, 5, 7, 10, 11, 13, 14 and 15 (Figure 1A). Gal-1 is a 14.5 kDa protein expressed in most mammalian organs, however, its expression pattern in the adult brain is limited to specific subtypes of astrocytes and neurons [215]. Gal-1 functions in at least three structural states: as a monomer, a dimer lectin and non-lectin, when disulfide bonds are formed [216]. When it acts as a lectin, it binds to glycans consisting of lactosamine sequences. Gal-1 functions both inside and outside cells, and is secreted using a non-canonical trans-golgi pathway.

### 7.1. Gal-1 Functions in the SVZ

It has been suggested that Gal-1 is expressed in the adult SVZ NSCs [215]. The Gal-1 expressing cells are sparsely distributed amongst GFAP+ SVZ astrocytes, but not in nearby striatal non-neurogenic astrocytes. Adult SVZ NSCs are slowly dividing, therefore, the adult NSC population is enriched with BrdU label-retaining astrocytes [217]. Interestingly, Gal-1 is expressed in a subset of these BrdU-label retaining cells, but not in the other SVZ cells, suggesting selective expression of Gal-1 in adult SVZ NSCs [215,218].

We have compared the lectin and non-lectin forms of Gal-1 by infusing their recombinant proteins into mouse LVs and found that only the lectin-form of Gal-1 increased the number of SVZ progenitor cells [215]. Infusion of Gal-1 and anti-Gal-1 neutralizing antibodies increased and decreased the number of BrdU-label retaining cells, respectively, suggesting that the number of the adult SVZ NSCs is positively regulated by the lectin form of Gal-1. In addition, infusion of Gal-1 protein or knocking down the *LGals-1* gene consistently increased and decreased the number of transit amplifying progenitors (TAP) cells, respectively [215]. However, it remained unclear whether this effect is due to direct binding of Gal-1 to TAP cells or through regulation of SVZ neural stem cells, which produce TAPS.

To examine which SVZ cell types the lectin form of Gal-1 binds in the brain, a biotinylated lectin form of Gal-1 was applied to adult brain sections [215]. Gal-1 binding was found on TAPS, neuroblasts, and sometimes on stem cells. On the other hand, mass spectrometric analysis of SVZ tissues using affinity columns showed that Gal-1 binds the β1 subunit of integrin (β1-Integrin). Infusion of β1-Integrin neutralizing antibody blocked Gal-1-induced increases in the number of BrdU label-retaining SVZ cells. β1-integrin plays critical roles in cell adhesion and is expressed by GFAP+ stem cells and DCX+ neuroblasts. Indeed, in vitro analysis using SVZ neurospheres revealed that Gal-1 regulates adhesion in concert with β1-integrin [219]. Finally, we analyzed downstream regulators of β1-integrin on SVZ adhesion by quantifying Akt phosphorylation. Akt Ser473 phosphorylation in a subset of SVZ astrocytes was significantly reduced in *Gal-1*^−/−^ mice, suggesting Gal-1 is needed for cell-adhesion mediated SVZ signaling [219]. Taken together, these results suggest that Gal-1 regulates SVZ NSC proliferation through regulating cell adhesion via binding carbohydrate structures on β1-Integrin.

### 7.2. Gal-1 Function in the Diseased Brain

Gal-1 function in the injured brain was examined using mouse models of brain ischemia [220] and spinal cord injury [221]. Gal-1 expression increased markedly in SVZ and peri-lesion striatal astrocytes in the ischemia model. As discussed above, brain ischemia increases SVZ neurogenesis and ectopic neuroblast migration to damaged brain regions. Interestingly, both neurogenesis and ectopic neuroblast migration were blocked by anti-Gal-1 neutralizing antibodies but enhanced by infusion of the lectin-form of Gal-1, but not by the non-lectin form [220]. Importantly, the Gal-1 lectin improved recovery from ischemia-induced functional deficits. We have also shown Gal-1 ameliorates functional recovery from spinal cord injury models in rodents and in the common marmoset [222]. Gal-1 expression is ubiquitous outside the CNS so it may be difficult to specifically pharmacologically target Gal-1 in the CNS. Nevertheless, it may be possible to target specific carbohydrate structures to which Gal-1 binds on NSCs or reactive astrocytes.

### 7.3. Gal-1 Regulates Hippocampal Neurogenesis

Immunohistochemical and morphological studies revealed that Gal-1 is expressed in a subset of DG NSCs and their immediate descendants, transit amplifying progenitor cells [218]. Histological analysis of *Gal-1*^−/−^ mutant mice injected with BrdU revealed that Gal-1 inhibits early stem and progenitor SGZ proliferation, however other work suggests it increases kainite induced SGZ neurogenesis [223]. Indeed, the number of adult DG granular neurons was increased in *Gal-1*^−/−^ mice. The mutant mice showed deficits in two types of hippocampal memory tests with normal level of anxiety [224]. The memory deficits could be explained by DG adult-neurogenesis functions in forgetting [225], although it is unknown whether the increased adult DG neurons in the mutant mice is the direct cause or not for the observed behavioral phenotypes. Further analysis by conditionally removing Gal-1 from NSCs may provide further insights.

## 8. Conclusions

The data discussed above show the powerful and broad effects that Gal-3, and to a lesser extent, Gal-1 has in brain health and disease. Remarkably, despite multiple binding partners, dozens of signaling targets and myriad intra- and extra-cellular functions, Gal-3 has very specific context-dependent effects. For example, it regulates neuroblast migration in the adult SVZ and in the postnatal niche it only regulates gliogenesis. Another example is that Gal-3 is necessary for neovascularization in the penumbra of stroke but not in the SVZ. How can this be? Theoretically Gal-3 loss or overexpression should be promiscuous, causing a complex set of cellular effects. Gal-3’s specialized functions are all the more unexpected since its loss results in an array of downstream molecular events both in homeostasis and in disease. One mechanism which could explain divergent cellular mechanisms of action in the SVZ is redistribution of its cellular pattern of expression. However, irrespective of the model of disease, Gal-3 cell type expression remains remarkably stable in the SVZ neurogenic niche. As for the parenchyma, where Gal-3 expression is usually undetectable with immunohistochemistry, it typically increases in microglia and in astrocytes in pathological contexts.

Whilst SVZ Gal-3 function in health and disease has been studied, its function in the SGZ of the hippocampal dentate gyrus remains obscure. Whether Gal-3 increases in the DG after injury is poorly explored. We speculate that Gal-3 immune barrier functions in the SVZ contribute to its expression. Gal-3 and Gal-1 have overlapping functions in adult neurogenesis but with some distinctions. They share several binding partners and general functions. It may well be that their co-expression in the niche helps to balance proliferation versus migration in adult homeostasis.

We have learned much about Gal-3 function in neurogenesis and brain pathology, but several important questions remain. It is unclear how Gal-3 expression in the SVZ influences the resting semi-inflamed status of the adult SVZ. We showed that modulation of Gal-3 levels in healthy neonates does not seem to modify inflammation [7], however it is not clear if this is the case in adults. It may be that Gal-3 expression in the SVZ is upregulated by inflammatory cytokines in the niche thus creating a feedforward cycle. In general more needs to be learned about how Gal-3 is regulated. Although Gal-3 is typically upregulated in brain injury and disease, we found that it was decreased in the SVZ in the cuprizone model of MS. What were the upstream mechanisms that caused this? Another unanswered question is how Gal-3 regulates signaling pathways within different cells of the SVZ. We found that both BMP and Wnt signaling were regulated by Gal-3 in the same SVZ cells. How is this coordinated? The new floxed Gal-3 mouse from EUCOMM, [7] will help dissect some of these issues. Use of scRNAseq to compare transcriptomics of Gal-3 loss-of-function to baseline will help dissect the role of Gal-3 at different stages of the lineage.

## Supplementary Materials

The following are available online at https://www.mdpi.com/article/10.3390/cells10113047/s1, Table S1: Selected Gal-3 binding partner functions and pathways; Table S2: Chemokine and cytokine mRNA changes in the SVZ after TMEV infection; Table S3: Angiogenesis relevant proteins altered by Gal-3.

## References

[1] Hirabayashi J., Kasai K. (1993). The family of metazoan metal-independent beta-galactoside-binding lectins: Structure, function and molecular evolution. Glycobiology.

[2] Starossom S.C., Mascanfroni I.D., Imitola J., Cao L., Raddassi K., Hernandez S.F., Bassil R., Croci D.O., Cerliani J.P., Delacour D. (2012). Galectin-1 deactivates classically activated microglia and protects from inflammation-induced neurodegeneration. Immunity.

[3] Li P., Liu S., Lu M., Bandyopadhyay G., Oh D., Imamura T., Johnson A.M., Sears D., Shen Z., Cui B. (2016). Hematopoietic-Derived Galectin-3 Causes Cellular and Systemic Insulin Resistance. Cell.

[4] Varki A., Cummings R.D., Esko J.D., Stanley P., Hart G.W., Aebi M., Darvill A.G., Kinoshita T., Packer N.H., Prestegard J.H. (2015). Galectins. Essentials of Glycobiology.

[5] Ochieng J., Furtak V., Lukyanov P. (2002). Extracellular functions of galectin-3. Glycoconj. J..

[6] Tan Y., Zheng Y., Xu D., Sun Z., Yang H., Yin Q. (2021). Galectin-3: A key player in microglia-mediated neuroinflammation and Alzheimer’s disease. Cell. Biosci..

[7] Al-Dalahmah O., Campos Soares L., Nicholson J., Draijer S., Mundim M., Lu V.M., Sun B., Tyler T., Adorjan I., O’Neill E. (2020). Galectin-3 modulates postnatal subventricular zone gliogenesis. Glia.

[8] Walther M., Kuklinski S., Pesheva P., Guntinas-Lichius O., Angelov D.N., Neiss W.F., Asou H., Probstmeier R. (2000). Galectin-3 is upregulated in microglial cells in response to ischemic brain lesions, but not to facial nerve axotomy. J. Neurosci. Res..

[9] Akazawa C., Nakamura Y., Sango K., Horie H., Kohsaka S. (2004). Distribution of the galectin-1 mRNA in the rat nervous system: Its transient upregulation in rat facial motor neurons after facial nerve axotomy. Neuroscience.

[10] Young C.C., Al-Dalahmah O., Lewis N.J., Brooks K.J., Jenkins M.M., Poirier F., Buchan A.M., Szele F.G. (2014). Blocked angiogenesis in Galectin-3 null mice does not alter cellular and behavioral recovery after middle cerebral artery occlusion stroke. Neurobiol. Dis..

[11] Boza-Serrano A., Ruiz R., Sanchez-Varo R., Garcia-Revilla J., Yang Y., Jimenez-Ferrer I., Paulus A., Wennstrom M., Vilalta A., Allendorf D. (2019). Galectin-3, a novel endogenous TREM2 ligand, detrimentally regulates inflammatory response in Alzheimer’s disease. Acta Neuropathol..

[12] Burguillos M.A., Svensson M., Schulte T., Boza-Serrano A., Garcia-Quintanilla A., Kavanagh E., Santiago M., Viceconte N., Oliva-Martin M.J., Osman A.M. (2015). Microglia-Secreted Galectin-3 Acts as a Toll-like Receptor 4 Ligand and Contributes to Microglial Activation. Cell Rep..

[13] Van den Brule F.A., Buicu C., Sobel M.E., Liu F.T., Castronovo V. (1995). Galectin-3, a laminin binding protein, fails to modulate adhesion of human melanoma cells to laminin. Neoplasma.

[14] Kuwabara I., Liu F.T. (1996). Galectin-3 promotes adhesion of human neutrophils to laminin. J. Immunol..

[15] Hikita C., Vijayakumar S., Takito J., Erdjument-Bromage H., Tempst P., Al-Awqati Q. (2000). Induction of terminal differentiation in epithelial cells requires polymerization of hensin by galectin 3. J. Cell Biol..

[16] Ochieng J., Warfield P., Green-Jarvis B., Fentie I. (1999). Galectin-3 regulates the adhesive interaction between breast carcinoma cells and elastin. J. Cell. Biochem..

[17] Ochieng J., Leite-Browning M.L., Warfield P. (1998). Regulation of cellular adhesion to extracellular matrix proteins by galectin-3. Biochem. Biophys. Res. Commun..

[18] Probstmeier R., Montag D., Schachner M. (1995). Galectin-3, a beta-galactoside-binding animal lectin, binds to neural recognition molecules. J. Neurochem..

[19] Partridge E.A., Le Roy C., Di Guglielmo G.M., Pawling J., Cheung P., Granovsky M., Nabi I.R., Wrana J.L., Dennis J.W. (2004). Regulation of cytokine receptors by Golgi N-glycan processing and endocytosis. Science.

[20] Yao Y., Zhou L., Liao W., Chen H., Du Z., Shao C., Wang P., Ding K. (2019). HH1-1, a novel Galectin-3 inhibitor, exerts anti-pancreatic cancer activity by blocking Galectin-3/EGFR/AKT/FOXO3 signaling pathway. Carbohydr. Polym..

[21] Comte I., Kim Y., Young C.C., van der Harg J.M., Hockberger P., Bolam P.J., Poirier F., Szele F.G. (2011). Galectin-3 maintains cell motility from the subventricular zone to the olfactory bulb. J. Cell Sci..

[22] Markowska A.I., Jefferies K.C., Panjwani N. (2011). Galectin-3 protein modulates cell surface expression and activation of vascular endothelial growth factor receptor 2 in human endothelial cells. J. Biol. Chem..

[23] Medzihradszky K.F., Kaasik K., Chalkley R.J. (2015). Tissue-Specific Glycosylation at the Glycopeptide Level. Mol. Cell Proteom..

[24] Kim S.J., Lee H.W., Gu Kang H., La S.H., Choi I.J., Ro J.Y., Bresalier R.S., Song J., Chun K.H. (2014). Ablation of galectin-3 induces p27(KIP1)-dependent premature senescence without oncogenic stress. Cell Death Differ..

[25] Elad-Sfadia G., Haklai R., Balan E., Kloog Y. (2004). Galectin-3 augments K-Ras activation and triggers a Ras signal that attenuates ERK but not phosphoinositide 3-kinase activity. J. Biol. Chem..

[26] Yang R.Y., Hsu D.K., Liu F.T. (1996). Expression of galectin-3 modulates T-cell growth and apoptosis. Proc. Natl. Acad. Sci. USA.

[27] Shimura T., Takenaka Y., Tsutsumi S., Hogan V., Kikuchi A., Raz A. (2004). Galectin-3, a novel binding partner of beta-catenin. Cancer Res..

[28] Al-Dalahmah O., Nicholson J., Draijer S., Soares L.C., Szele F.G. (2020). Galectin-3 diminishes Wnt signaling in the postnatal subventricular zone. Stem Cells.

[29] Banfer S., Schneider D., Dewes J., Strauss M.T., Freibert S.A., Heimerl T., Maier U.G., Elsasser H.P., Jungmann R., Jacob R. (2018). Molecular mechanism to recruit galectin-3 into multivesicular bodies for polarized exosomal secretion. Proc. Natl. Acad. Sci. USA.

[30] Jia J., Claude-Taupin A., Gu Y., Choi S.W., Peters R., Bissa B., Mudd M.H., Allers L., Pallikkuth S., Lidke K.A. (2020). Galectin-3 Coordinates a Cellular System for Lysosomal Repair and Removal. Dev. Cell.

[31] Dumic J., Dabelic S., Flogel M. (2006). Galectin-3: An open-ended story. Biochim. Biophys. Acta.

[32] Srejovic I., Selakovic D., Jovicic N., Jakovljevic V., Lukic M.L., Rosic G. (2020). Galectin-3: Roles in Neurodevelopment, Neuroinflammation, and Behavior. Biomolecules.

[33] Johannes L., Jacob R., Leffler H. (2018). Galectins at a glance. J. Cell Sci..

[34] Goings G.E., Kozlowski D.A., Szele F.G. (2006). Differential activation of microglia in neurogenic versus non-neurogenic regions of the forebrain. Glia.

[35] Bardella C., Al-Shammari A.R., Soares L., Tomlinson I., O’Neill E., Szele F.G. (2018). The role of inflammation in subventricular zone cancer. Prog. Neurobiol..

[36] Altman J. (1969). Autoradiographic and histological studies of postnatal neurogenesis. IV. Cell proliferation and migration in the anterior forebrain, with special reference to persisting neurogenesis in the olfactory bulb. J. Comp. Neurol..

[37] Altman J. (1962). Are new neurons formed in the brains of adult mammals?. Science.

[38] Vilar M., Mira H. (2016). Regulation of Neurogenesis by Neurotrophins during Adulthood: Expected and Unexpected Roles. Front. Neurosci..

[39] Faigle R., Song H. (2013). Signaling mechanisms regulating adult neural stem cells and neurogenesis. Biochim. Biophys. Acta.

[40] Fuentealba L.C., Obernier K., Alvarez-Buylla A. (2012). Adult neural stem cells bridge their niche. Cell Stem Cell.

[41] Doetsch F., Garcia-Verdugo J.M., Alvarez-Buylla A. (1997). Cellular composition and three-dimensional organization of the subventricular germinal zone in the adult mammalian brain. J. Neurosci..

[42] Gheusi G., Lledo P.M. (2014). Adult neurogenesis in the olfactory system shapes odor memory and perception. Prog. Brain. Res..

[43] Kalamakis G., Brune D., Ravichandran S., Bolz J., Fan W., Ziebell F., Stiehl T., Catala-Martinez F., Kupke J., Zhao S. (2019). Quiescence Modulates Stem Cell Maintenance and Regenerative Capacity in the Aging Brain. Cell.

[44] Dulken B.W., Leeman D.S., Boutet S.C., Hebestreit K., Brunet A. (2017). Single-Cell Transcriptomic Analysis Defines Heterogeneity and Transcriptional Dynamics in the Adult Neural Stem Cell Lineage. Cell Rep..

[45] Mizrak D., Levitin H.M., Delgado A.C., Crotet V., Yuan J., Chaker Z., Silva-Vargas V., Sims P.A., Doetsch F. (2019). Single-Cell Analysis of Regional Differences in Adult V-SVZ Neural Stem Cell Lineages. Cell Rep..

[46] Hochgerner H., Zeisel A., Lonnerberg P., Linnarsson S. (2018). Conserved properties of dentate gyrus neurogenesis across postnatal development revealed by single-cell RNA sequencing. Nat. Neurosci..

[47] Shin J., Berg D.A., Zhu Y., Shin J.Y., Song J., Bonaguidi M.A., Enikolopov G., Nauen D.W., Christian K.M., Ming G.L. (2015). Single-Cell RNA-Seq with Waterfall Reveals Molecular Cascades underlying Adult Neurogenesis. Cell Stem Cell.

[48] Moreno-Valladares M., Moreno-Cugnon L., Silva T.M., Garces J.P., Saenz-Antonanzas A., Alvarez-Satta M., Matheu A. (2020). CD8(+) T cells are increased in the subventricular zone with physiological and pathological aging. Aging Cell.

[49] Dulken B.W., Buckley M.T., Navarro Negredo P., Saligrama N., Cayrol R., Leeman D.S., George B.M., Boutet S.C., Hebestreit K., Pluvinage J.V. (2019). Single-cell analysis reveals T cell infiltration in old neurogenic niches. Nature.

[50] James R.E., Hillis J., Adorjan I., Gration B., Mundim M.V., Iqbal A.J., Majumdar M.M., Yates R.L., Richards M.M., Goings G.E. (2016). Loss of galectin-3 decreases the number of immune cells in the subventricular zone and restores proliferation in a viral model of multiple sclerosis. Glia.

[51] Gonçalves J.T., Schafer S.T., Gage F.H. (2016). Adult Neurogenesis in the Hippocampus: From Stem Cells to Behavior. Cell.

[52] Moreno-Jiménez E.P., Flor-García M., Terreros-Roncal J., Rábano A., Cafini F., Pallas-Bazarra N., Ávila J., Llorens-Martín M. (2019). Adult hippocampal neurogenesis is abundant in neurologically healthy subjects and drops sharply in patients with Alzheimer’s disease. Nat. Med..

[53] Ernst A., Alkass K., Bernard S., SalSehpour M., Perl S., Tisdale J., Possnert G., Druid H., Frisen J. (2014). Neurogenesis in the striatum of the adult human brain. Cell.

[54] Paredes M.F., James D., Gil-Perotin S., Kim H., Cotter J.A., Ng C., Sandoval K., Rowitch D.H., Xu D., McQuillen P.S. (2016). Extensive migration of young neurons into the infant human frontal lobe. Science.

[55] Sanai N., Nguyen T., Ihrie R.A., Mirzadeh Z., Tsai H.H., Wong M., Gupta N., Berger M.S., Huang E., Garcia-Verdugo J.M. (2011). Corridors of migrating neurons in the human brain and their decline during infancy. Nature.

[56] Sorrells S.F., Paredes M.F., Cebrian-Silla A., Sandoval K., Qi D., Kelley K.W., James D., Mayer S., Chang J., Auguste K.I. (2018). Human hippocampal neurogenesis drops sharply in children to undetectable levels in adults. Nature.

[57] Young C.C., van der Harg J.M., Lewis N.J., Brooks K.J., Buchan A.M., Szele F.G. (2013). Ependymal ciliary dysfunction and reactive astrocytosis in a reorganized subventricular zone after stroke. Cereb. Cortex.

[58] Hillis J.M., Davies J., Mundim M.V., Al-Dalahmah O., Szele F.G. (2016). Cuprizone demyelination induces a unique inflammatory response in the subventricular zone. J. Neuroinflammation.

[59] Zywitza V., Misios A., Bunatyan L., Willnow T.E., Rajewsky N. (2018). Single-Cell Transcriptomics Characterizes Cell Types in the Subventricular Zone and Uncovers Molecular Defects Impairing Adult Neurogenesis. Cell Rep..

[60] Kim Y., Comte I., Szabo G., Hockberger P., Szele F.G. (2009). Adult mouse subventricular zone stem and progenitor cells are sessile and epidermal growth factor receptor negatively regulates neuroblast migration. PLoS ONE.

[61] Levison S.W., Goldman J.E. (1993). Both oligodendrocytes and astrocytes develop from progenitors in the subventricular zone of postnatal rat forebrain. Neuron.

[62] Sirko S., Irmler M., Gascon S., Bek S., Schneider S., Dimou L., Obermann J., De Souza Paiva D., Poirier F., Beckers J. (2015). Astrocyte reactivity after brain injury-: The role of galectins 1 and 3. Glia.

[63] Lalancette-Hebert M., Swarup V., Beaulieu J.M., Bohacek I., Abdelhamid E., Weng Y.C., Sato S., Kriz J. (2012). Galectin-3 is required for resident microglia activation and proliferation in response to ischemic injury. J. Neurosci..

[64] Siew J.J., Chen H.M., Chen H.Y., Chen H.L., Chen C.M., Soong B.W., Wu Y.R., Chang C.P., Chan Y.C., Lin C.H. (2019). Galectin-3 is required for the microglia-mediated brain inflammation in a model of Huntington’s disease. Nat. Commun..

[65] Qu Q., Sun G., Li W., Yang S., Ye P., Zhao C., Yu R.T., Gage F.H., Evans R.M., Shi Y. (2010). Orphan nuclear receptor TLX activates Wnt/beta-catenin signalling to stimulate neural stem cell proliferation and self-renewal. Nat. Cell Biol..

[66] Piccin D., Morshead C.M. (2011). Wnt signaling regulates symmetry of division of neural stem cells in the adult brain and in response to injury. Stem Cells.

[67] Adachi K., Mirzadeh Z., Sakaguchi M., Yamashita T., Nikolcheva T., Gotoh Y., Peltz G., Gong L., Kawase T., Alvarez-Buylla A. (2007). Beta-catenin signaling promotes proliferation of progenitor cells in the adult mouse subventricular zone. Stem Cells.

[68] Azim K., Fischer B., Hurtado-Chong A., Draganova K., Cantu C., Zemke M., Sommer L., Butt A., Raineteau O. (2014). Persistent Wnt/beta-Catenin signaling determines dorsalization of the postnatal subventricular zone and neural stem cell specification into oligodendrocytes and glutamatergic neurons. Stem Cells.

[69] Marinaro C., Pannese M., Weinandy F., Sessa A., Bergamaschi A., Taketo M.M., Broccoli V., Comi G., Gotz M., Martino G. (2012). Wnt signaling has opposing roles in the developing and the adult brain that are modulated by Hipk1. Cereb. Cortex.

[70] Kobayashi T., Shimura T., Yajima T., Kubo N., Araki K., Tsutsumi S., Suzuki H., Kuwano H., Raz A. (2011). Transient gene silencing of galectin-3 suppresses pancreatic cancer cell migration and invasion through degradation of beta-catenin. Int. J. Cancer J. Int. Du Cancer.

[71] Song S., Mazurek N., Liu C., Sun Y., Ding Q.Q., Liu K., Hung M.C., Bresalier R.S. (2009). Galectin-3 mediates nuclear beta-catenin accumulation and Wnt signaling in human colon cancer cells by regulation of glycogen synthase kinase-3beta activity. Cancer Res..

[72] Zhang D., Chen Z.G., Liu S.H., Dong Z.Q., Dalin M., Bao S.S., Hu Y.W., Wei F.C. (2013). Galectin-3 gene silencing inhibits migration and invasion of human tongue cancer cells in vitro via downregulating beta-catenin. Acta Pharm. Sin..

[73] Colak D., Mori T., Brill M.S., Pfeifer A., Falk S., Deng C., Monteiro R., Mummery C., Sommer L., Gotz M. (2008). Adult neurogenesis requires Smad4-mediated bone morphogenic protein signaling in stem cells. J. Neurosci..

[74] Lim D.A., Tramontin A.D., Trevejo J.M., Herrera D.G., Garcia-Verdugo J.M., Alvarez-Buylla A. (2000). Noggin antagonizes BMP signaling to create a niche for adult neurogenesis. Neuron.

[75] Gajera C.R., Emich H., Lioubinski O., Christ A., Beckervordersandforth-Bonk R., Yoshikawa K., Bachmann S., Christensen E.I., Gotz M., Kempermann G. (2010). LRP2 in ependymal cells regulates BMP signaling in the adult neurogenic niche. J. Cell Sci..

[76] Mercier F., Douet V. (2014). Bone morphogenetic protein-4 inhibits adult neurogenesis and is regulated by fractone-associated heparan sulfates in the subventricular zone. J. Chem. Neuroanat..

[77] Bonaguidi M.A., McGuire T., Hu M., Kan L., Samanta J., Kessler J.A. (2005). LIF and BMP signaling generate separate and discrete types of GFAP-expressing cells. Development.

[78] Ciceroni C., Mosillo P., Mastrantoni E., Sale P., Ricci-Vitiani L., Biagioni F., Stocchi F., Nicoletti F., Melchiorri D. (2010). mGLU3 metabotropic glutamate receptors modulate the differentiation of SVZ-derived neural stem cells towards the astrocytic lineage. Glia.

[79] Gomes W.A., Mehler M.F., Kessler J.A. (2003). Transgenic overexpression of BMP4 increases astroglial and decreases oligodendroglial lineage commitment. Dev. Biol..

[80] Grinspan J.B., Edell E., Carpio D.F., Beesley J.S., Lavy L., Pleasure D., Golden J.A. (2000). Stage-specific effects of bone morphogenetic proteins on the oligodendrocyte lineage. J. Neurobiol..

[81] Hegarty S.V., O’Keeffe G.W., Sullivan A.M. (2013). BMP-Smad 1/5/8 signalling in the development of the nervous system. Prog. Neurobiol..

[82] Hanahan D., Weinberg R.A. (2011). Hallmarks of Cancer: The Next Generation. Cell.

[83] Colotta F., Allavena P., Sica A., Garlanda C., Mantovani A. (2009). Cancer-related inflammation, the seventh hallmark of cancer: Links to genetic instability. Carcinogenesis.

[84] Dvorak H.F. (1986). Tumors: Wounds That Do Not Heal. New Engl. J. Med..

[85] Grivennikov S.I., Greten F.R., Karin M. (2010). Immunity, inflammation, and cancer. Cell.

[86] Qian B.-Z., Pollard J.W. (2010). Macrophage Diversity Enhances Tumor Progression and Metastasis. Cell.

[87] Sowers J.L., Johnson K.M., Conrad C., Patterson J.T., Sowers L.C. (2014). The role of inflammation in brain cancer. Adv. Exp. Med. Biol..

[88] Hussain S.P., Hofseth L.J., Harris C.C. (2003). Radical causes of cancer. Nat. Rev. Cancer.

[89] Arnold K.M., Opdenaker L.M., Flynn D., Sims-Mourtada J. (2015). Wound healing and cancer stem cells: Inflammation as a driver of treatment resistance in breast cancer. Cancer Growth Metastasis.

[90] Jeon S.B., Yoon H.J., Chang C.Y., Koh H.S., Jeon S.H., Park E.J. (2010). Galectin-3 exerts cytokine-like regulatory actions through the JAK-STAT pathway. J. Immunol..

[91] Neder L., Marie S.K., Carlotti C.G., Gabbai A.A., Rosemberg S., Malheiros S.M., Siqueira R.P., Oba-Shinjo S.M., Uno M., Aguiar P.H. (2004). Galectin-3 as an immunohistochemical tool to distinguish pilocytic astrocytomas from diffuse astrocytomas, and glioblastomas from anaplastic oligodendrogliomas. Brain Pathol..

[92] Strik H.M., Deininger M.H., Frank B., Schluesener H.J., Meyermann R. (2001). Galectin-3: Cellular Distribution and Correlation with WHO-grade in Human Gliomas. J. Neuro-Oncol..

[93] Deininger M.H., Trautmann K., Meyermann R., Schluesener H.J. (2002). Galectin-3 labeling correlates positively in tumor cells and negatively in endothelial cells with malignancy and poor prognosis in oligodendroglioma patients. Anticancer Res..

[94] Chen A., Jiang Y., Li Z., Wu L., Santiago U., Zou H., Cai C., Sharma V., Guan Y., McCarl L.H. (2021). Chitinase-3-like 1 protein complexes modulate macrophage-mediated immune suppression in glioblastoma. J. Clin. Investig..

[95] Akahani S., Nangia-Makker P., Inohara H., Kim H.R., Raz A. (1997). Galectin-3: A novel antiapoptotic molecule with a functional BH1(NWGR) domain of Bcl-2 family. Cancer Res..

[96] Fukumori T., Takenaka Y., Oka N., Yoshii T., Hogan V., Inohara H., Kanayama H.-O., Kim H.-R.C., Raz A. (2004). Endogenous galectin-3 determines the routing of CD95 apoptotic signaling pathways. Cancer Res..

[97] Liu L., Sakai T., Sano N., Fukui K. (2004). Nucling mediates apoptosis by inhibiting expression of galectin-3 through interference with nuclear factor kappaB signalling. Biochem. J..

[98] Yu F., Finley R.L., Raz A., Kim H.-R.C. (2002). Galectin-3 translocates to the perinuclear membranes and inhibits cytochrome c release from the mitochondria A role for synexin in galectin-3 translocation. J. Biol. Chem..

[99] Kim H.-R.C., Lin H.-M., Biliran H., Raz A. (1999). Cell Cycle Arrest and Inhibition of Anoikis by Galectin-3 in Human Breast Epithelial Cells. Cancer Res..

[100] Zhao Q., Barclay M., Hilkens J., Guo X., Barrow H., Rhodes J.M., Yu L.-G. (2010). Interaction between circulating galectin-3 and cancer-associated MUC1 enhances tumour cell homotypic aggregation and prevents anoikis. Mol. Cancer.

[101] Seguin L., Kato S., Franovic A., Camargo M.F., Lesperance J., Elliott K.C., Yebra M., Mielgo A., Lowy A.M., Husain H. (2014). An integrin β₃-KRAS-RalB complex drives tumour stemness and resistance to EGFR inhibition. Nat. Cell Biol..

[102] Seguin L., Desgrosellier J.S., Weis S.M., Cheresh D.A. (2015). Integrins and cancer: Regulators of cancer stemness, metastasis, and drug resistance. Trends Cell Biol..

[103] Seguin L., Camargo M.F., Wettersten H.I., Kato S., Desgrosellier J.S., von Schalscha T., Elliott K.C., Cosset E., Lesperance J., Weis S.M. (2017). Galectin-3, a Druggable Vulnerability for KRAS-Addicted Cancers. Cancer Discov..

[104] Steed T.C., Treiber J.M., Patel K., Ramakrishnan V., Merk A., Smith A.R., Carter B.S., Dale A.M., Chow L.M., Chen C.C. (2016). Differential localization of glioblastoma subtype: Implications on glioblastoma pathogenesis. Oncotarget.

[105] Sanai N., Alvarez-Buylla A., Berger M.S. (2005). Neural Stem Cells and the Origin of Gliomas. New Engl. J. Med..

[106] Lim D.A., Cha S., Mayo M.C., Chen M.-H., Keles E., VandenBerg S., Berger M.S. (2007). Relationship of glioblastoma multiforme to neural stem cell regions predicts invasive and multifocal tumor phenotype. Neuro-Oncology.

[107] Liu S., Wang Y., Fan X., Ma J., Ma W., Wang R., Jiang T. (2016). Anatomical Involvement of the Subventricular Zone Predicts Poor Survival Outcome in Low-Grade Astrocytomas. PLoS ONE.

[108] Liu S., Wang Y., Fan X., Ma J., Qiu X., Jiang T. (2017). Association of MRI-classified subventricular regions with survival outcomes in patients with anaplastic glioma. Clin. Radiol..

[109] Chen L., Chaichana K.L., Kleinberg L., Ye X., Quinones-Hinojosa A., Redmond K. (2015). Glioblastoma recurrence patterns near neural stem cell regions. Radiother. Oncol..

[110] Jafri N.F., Clarke J.L., Weinberg V., Barani I.J., Cha S. (2013). Relationship of glioblastoma multiforme to the subventricular zone is associated with survival. Neuro. Oncol..

[111] Mistry A.M., Hale A.T., Chambless L.B., Weaver K.D., Thompson R.C., Ihrie R.A. (2017). Influence of glioblastoma contact with the lateral ventricle on survival: A meta-analysis. J. Neurooncol..

[112] Vergani F., Martino J., Goze C., Rigau V., Duffau H. (2011). World Health Organization Grade II gliomas and subventricular zone: Anatomic, genetic, and clinical considerations. Neurosurgery.

[113] Young G.S., Macklin E.A., Setayesh K., Lawson J.D., Wen P.Y., Norden A.D., Drappatz J., Kesari S. (2011). Longitudinal MRI evidence for decreased survival among periventricular glioblastoma. J. Neurooncol..

[114] Adeberg S., König L., Bostel T., Harrabi S., Welzel T., Debus J., Combs S.E. (2014). Glioblastoma Recurrence Patterns After Radiation Therapy With Regard to the Subventricular Zone. Int. J. Radiat. Oncol. Biol. Phys..

[115] Gupta T., Nair V., Jalali R. (2014). Stem cell niche irradiation in glioblastoma: Providing a ray of hope?. CNS Oncol..

[116] Verhaak R.G.W., Hoadley K.A., Purdom E., Wang V., Qi Y., Wilkerson M.D., Miller C.R., Ding L., Golub T., Mesirov J.P. (2010). Integrated Genomic Analysis Identifies Clinically Relevant Subtypes of Glioblastoma Characterized by Abnormalities in PDGFRA, IDH1, EGFR, and NF1. Cancer Cell.

[117] Seguin L., Odouard S., Corlazzoli F., Haddad S.A., Moindrot L., Calvo Tardon M., Yebra M., Koval A., Marinari E., Bes V. (2021). Macropinocytosis requires Gal-3 in a subset of patient-derived glioblastoma stem cells. Commun. Biol..

[118] Nakajima K., Kho D.H., Yanagawa T., Harazono Y., Gao X., Hogan V., Raz A. (2014). Galectin-3 Inhibits Osteoblast Differentiation through Notch Signaling. Neoplasia.

[119] Kang H.G., Kim D.-H., Kim S.-J., Cho Y., Jung J., Jang W., Chun K.-H. (2016). Galectin-3 supports stemness in ovarian cancer stem cells by activation of the Notch1 intracellular domain. Oncotarget.

[120] Kuo H.Y., Hsu H.T., Chen Y.C., Chang Y.W., Liu F.T., Wu C.W. (2016). Galectin-3 modulates the EGFR signalling-mediated regulation of Sox2 expression via c-Myc in lung cancer. Glycobiology.

[121] Bazzoni R., Bentivegna A. (2019). Role of Notch Signaling Pathway in Glioblastoma Pathogenesis. Cancers.

[122] Stockhausen M.-T., Kristoffersen K., Poulsen H.S. (2010). The functional role of Notch signaling in human gliomas. Neuro. Oncol..

[123] Xu H., Zong H., Ma C., Ming X., Shang M., Li K., He X., Du H., Cao L. (2017). Epidermal growth factor receptor in glioblastoma. Oncol. Lett..

[124] Hatanpaa K.J., Burma S., Zhao D., Habib A.A. (2010). Epidermal growth factor receptor in glioma: Signal transduction, neuropathology, imaging, and radioresistance. Neoplasia.

[125] Dos Santos S.N., Sheldon H., Pereira J.X., Paluch C., Bridges E.M., El-Cheikh M.C., Harris A.L., Bernardes E.S. (2017). Galectin-3 acts as an angiogenic switch to induce tumor angiogenesis via Jagged-1/Notch activation. Oncotarget.

[126] Xi G., Best B., Mania-Farnell B., James C.D., Tomita T. (2017). Therapeutic Potential for Bone Morphogenetic Protein 4 in Human Malignant Glioma. Neoplasia.

[127] Sachdeva R., Wu M., Johnson K., Kim H., Celebre A., Shahzad U., Graham M.S., Kessler J.A., Chuang J.H., Karamchandani J. (2019). BMP signaling mediates glioma stem cell quiescence and confers treatment resistance in glioblastoma. Sci. Rep..

[128] He L., Zhou H., Zeng Z., Yao H., Jiang W., Qu H. (2019). Wnt/β-catenin signaling cascade: A promising target for glioma therapy. J. Cell. Physiol..

[129] Shimura T., Takenaka Y., Fukumori T., Tsutsumi S., Okada K., Hogan V., Kikuchi A., Kuwano H., Raz A. (2005). Implication of galectin-3 in Wnt signaling. Cancer Res..

[130] Bardella C., Al-Dalahmah O., Krell D., Brazauskas P., Al-Qahtani K., Tomkova M., Adam J., Serres S., Lockstone H., Freeman-Mills L. (2016). Expression of Idh1R132H in the Murine Subventricular Zone Stem Cell Niche Recapitulates Features of Early Gliomagenesis. Cancer Cell.

[131] Liu F.T., Rabinovich G.A. (2005). Galectins as modulators of tumour progression. Nat. Rev. Cancer.

[132] Fortuna-Costa A., Gomes A.M., Kozlowski E.O., Stelling M.P., Pavão M.S.G. (2014). Extracellular Galectin-3 in Tumor Progression and Metastasis. Front. Oncol..

[133] Yamamoto H., Swoger J., Greene S., Saito T., Hurh J., Sweeley C., Leestma J., Mkrdichian E., Cerullo L., Nishikawa A. (2000). β1,6-N-Acetylglucosamine-bearing N-Glycans in Human Gliomas: Implications for a Role in Regulating Invasivity. Cancer Res..

[134] Padhiar A.A., Fan J., Tang Y., Yu J., Wang S., Liu L., Niang B., Annani-Akollor M.E., Wang L., Wang Q. (2015). Upregulated β1-6 branch N-glycan marks early gliomagenesis but exhibited biphasic expression in the progression of astrocytic glioma. Am. J. Cancer Res..

[135] Hassani Z., Saleh A., Turpault S., Khiati S., Morelle W., Vignon J., Hugnot J.P., Uro-Coste E., Legrand P., Delaforge M. (2017). Phostine PST3.1a Targets MGAT5 and Inhibits Glioblastoma-Initiating Cell Invasiveness and Proliferation. Mol. Cancer Res..

[136] Wang H., Song X., Huang Q., Xu T., Yun D., Wang Y., Hu L., Yan Y., Chen H., Lu D. (2019). LGALS3 Promotes Treatment Resistance in Glioblastoma and Is Associated with Tumor Risk and Prognosis. Cancer Epidemiol. Biomark. Prev..

[137] Wdowiak K., Francuz T., Gallego-Colon E., Ruiz-Agamez N., Kubeczko M., Grochoła I., Wojnar J. (2018). Galectin Targeted Therapy in Oncology: Current Knowledge and Perspectives. Int. J. Mol. Sci..

[138] Stegmayr J., Zetterberg F., Carlsson M.C., Huang X., Sharma G., Kahl-Knutson B., Schambye H., Nilsson U.J., Oredsson S., Leffler H. (2019). Extracellular and intracellular small-molecule galectin-3 inhibitors. Sci. Rep..

[139] Compston A., Coles A. (2008). Multiple sclerosis. Lancet.

[140] Denic A., Johnson A.J., Bieber A.J., Warrington A.E., Rodriguez M., Pirko I. (2011). The relevance of animal models in multiple sclerosis research. Pathophysiology.

[141] Xing Y.L., Roth P.T., Stratton J.A., Chuang B.H., Danne J., Ellis S.L., Ng S.W., Kilpatrick T.J., Merson T.D. (2014). Adult neural precursor cells from the subventricular zone contribute significantly to oligodendrocyte regeneration and remyelination. J. Neurosci..

[142] Nait-Oumesmar B., Decker L., Lachapelle F., Avellana-Adalid V., Bachelin C., Van Evercooren A.B. (1999). Progenitor cells of the adult mouse subventricular zone proliferate, migrate and differentiate into oligodendrocytes after demyelination. Eur. J. Neurosci..

[143] Nait-Oumesmar B., Picard-Riera N., Kerninon C., Decker L., Seilhean D., Hoglinger G.U., Hirsch E.C., Reynolds R., Baron-Van Evercooren A. (2007). Activation of the subventricular zone in multiple sclerosis: Evidence for early glial progenitors. Proc. Natl. Acad. Sci. USA.

[144] Picard-Riera N., Decker L., Delarasse C., Goude K., Nait-Oumesmar B., Liblau R., Pham-Dinh D., Evercooren A.B. (2002). Experimental autoimmune encephalomyelitis mobilizes neural progenitors from the subventricular zone to undergo oligodendrogenesis in adult mice. Proc. Natl. Acad. Sci. USA.

[145] Esposito F., Wojcik J., Rodegher M., Radaelli M., Moiola L., Ghezzi A., Capra R., Brambilla P., Sorosina M., Giacalone G. (2011). MGAT5 and disease severity in progressive multiple sclerosis. J. Neuroimmunol..

[146] Lee S.U., Grigorian A., Pawling J., Chen I.J., Gao G., Mozaffar T., McKerlie C., Demetriou M. (2007). N-glycan processing deficiency promotes spontaneous inflammatory demyelination and neurodegeneration. J. Biol. Chem..

[147] Stancic M., van Horssen J., Thijssen V.L., Gabius H.J., van der Valk P., Hoekstra D., Baron W. (2011). Increased expression of distinct galectins in multiple sclerosis lesions. Neuropathol. Appl. Neurobiol..

[148] Haines J.D., Vidaurre O.G., Zhang F., Riffo-Campos A.L., Castillo J., Casanova B., Casaccia P., Lopez-Rodas G. (2015). Multiple sclerosis patient-derived CSF induces transcriptional changes in proliferating oligodendrocyte progenitors. Mult. Scler..

[149] Dawson J.D. (1916). The histology of disseminated sclerosis. R. Soc. Edin..

[150] Reichert F., Rotshenker S. (1999). Galectin-3/MAC-2 in experimental allergic encephalomyelitis. Exp. Neurol..

[151] Goings G.E., Greisman A., James R.E., Abram L.K., Begolka W.S., Miller S.D., Szele F.G. (2008). Hematopoietic cell activation in the subventricular zone after Theiler’s virus infection. J. Neuroinflammation.

[152] Hoyos H.C., Rinaldi M., Mendez-Huergo S.P., Marder M., Rabinovich G.A., Pasquini J.M., Pasquini L.A. (2014). Galectin-3 controls the response of microglial cells to limit cuprizone-induced demyelination. Neurobiol. Dis..

[153] Jiang H.R., Al Rasebi Z., Mensah-Brown E., Shahin A., Xu D., Goodyear C.S., Fukada S.Y., Liu F.T., Liew F.Y., Lukic M.L. (2009). Galectin-3 Deficiency Reduces the Severity of Experimental Autoimmune Encephalomyelitis. J. Immunol..

[154] Rotshenker S., Reichert F., Gitik M., Haklai R., Elad-Sfadia G., Kloog Y. (2008). Galectin-3/MAC-2, Ras and PI3K activate complement receptor-3 and scavenger receptor-AI/II mediated myelin phagocytosis in microglia. Glia.

[155] Kim Y.S., Choi D.H., Block M.L., Lorenzl S., Yang L., Kim Y.J., Sugama S., Cho B.P., Hwang O., Browne S.E. (2007). A pivotal role of matrix metalloproteinase-3 activity in dopaminergic neuronal degeneration via microglial activation. FASEB J..

[156] Hoyos H.C., Marder M., Ulrich R., Gudi V., Stangel M., Rabinovich G.A., Pasquini L.A., Pasquini J.M. (2016). The Role of Galectin-3: From Oligodendroglial Differentiation and Myelination to Demyelination and Remyelination Processes in a Cuprizone-Induced Demyelination Model. Adv. Exp. Med. Biol..

[157] Espinosa-Oliva A.M., Garcia-Miranda P., Alonso-Bellido I.M., Carvajal A.E., Gonzalez-Rodriguez M., Carrillo-Jimenez A., Temblador A.J., Felices-Navarro M., Garcia-Dominguez I., Roca-Ceballos M.A. (2021). Galectin-3 Deletion Reduces LPS and Acute Colitis-Induced Pro-Inflammatory Microglial Activation in the Ventral Mesencephalon. Front. Pharmacol..

[158] Kebir H., Kreymborg K., Ifergan I., Dodelet-Devillers A., Cayrol R., Bernard M., Giuliani F., Arbour N., Becher B., Prat A. (2007). Human TH17 lymphocytes promote blood-brain barrier disruption and central nervous system inflammation. Nat. Med..

[159] Nishihara H., Shimizu F., Kitagawa T., Yamanaka N., Akada J., Kuramitsu Y., Sano Y., Takeshita Y., Maeda T., Abe M. (2017). Identification of galectin-3 as a possible antibody target for secondary progressive multiple sclerosis. Mult. Scler..

[160] Pasquini L.A., Millet V., Hoyos H.C., Giannoni J.P., Croci D.O., Marder M., Liu F.T., Rabinovich G.A., Pasquini J.M. (2011). Galectin-3 drives oligodendrocyte differentiation to control myelin integrity and function. Cell Death Differ..

[161] Young C.C., Brooks K.J., Buchan A.M., Szele F.G. (2011). Cellular and molecular determinants of stroke-induced changes in subventricular zone cell migration. Antioxid. Redox Signal.

[162] Dong H., Wang Z.H., Zhang N., Liu S.D., Zhao J.J., Liu S.Y. (2017). Serum Galectin-3 level, not Galectin-1, is associated with the clinical feature and outcome in patients with acute ischemic stroke. Oncotarget.

[163] He X.W., Li W.L., Li C., Liu P., Shen Y.G., Zhu M., Jin X.P. (2017). Serum levels of galectin-1, galectin-3, and galectin-9 are associated with large artery atherosclerotic stroke. Sci. Rep..

[164] Beck H., Plate K.H. (2009). Angiogenesis after cerebral ischemia. Acta Neuropathol..

[165] Zhang Z.G., Zhang L., Jiang Q., Zhang R., Davies K., Powers C., Bruggen N., Chopp M. (2000). VEGF enhances angiogenesis and promotes blood-brain barrier leakage in the ischemic brain. J. Clin. Investig..

[166] Zhuang J.J., Zhou L., Zheng Y.H., Ding Y.S. (2021). The serum galectin-3 levels are associated with the severity and prognosis of ischemic stroke. Aging.

[167] Fenton-Navarro B., Garduno Rios D., Torner L., Letechipia-Vallejo G., Cervantes M. (2021). Melatonin Decreases Circulating Levels of Galectin-3 and Cytokines, Motor Activity, and Anxiety Following Acute Global Cerebral Ischemia in Male Rats. Arch. Med. Res..

[168] Wesley U.V., Sutton I.C., Cunningham K., Jaeger J.W., Phan A.Q., Hatcher J.F., Dempsey R.J. (2021). Galectin-3 protects against ischemic stroke by promoting neuro-angiogenesis via apoptosis inhibition and Akt/Caspase regulation. J. Cereb. Blood Flow Metab..

[169] Doverhag C., Hedtjarn M., Poirier F., Mallard C., Hagberg H., Karlsson A., Savman K. (2010). Galectin-3 contributes to neonatal hypoxic-ischemic brain injury. Neurobiol. Dis..

[170] Lalancette-Hebert M., Gowing G., Simard A., Weng Y.C., Kriz J. (2007). Selective ablation of proliferating microglial cells exacerbates ischemic injury in the brain. J. Neurosci..

[171] Yan Y.P., Lang B.T., Vemuganti R., Dempsey R.J. (2009). Galectin-3 mediates post-ischemic tissue remodeling. Brain. Res..

[172] Savman K., Wang W., Rafati A.H., Svedin P., Nair S., Golubinskaya V., Ardalan M., Brown K.L., Karlsson-Bengtsson A., Mallard C. (2021). Galectin-3 Modulates Microglia Inflammation in vitro but Not Neonatal Brain Injury in vivo under Inflammatory Conditions. Dev. Neurosci..

[173] Da Rosa M.M., de Aguiar Ferreira M., de Oliveira Lima C.A., Santos Mendonca A.C., Silva Y.M., Sharjeel M., de Melo Rego M.J.B., Pereira M.C., da Rocha Pitta M.G. (2021). Alzheimer’s disease: Is there a role for galectins?. Eur. J. Pharm..

[174] Wang X., Zhang S., Lin F., Chu W., Yue S. (2013). Elevated Galectin-3 Levels in the Serum of Patients With Alzheimer’s Disease. Am. J. Alzheimer’s Dis. Other Dement..

[175] Yazar T., Olgun Yazar H., Cihan M. (2021). Evaluation of serum galectin-3 levels at Alzheimer patients by stages: A preliminary report. Acta Neurol. Belg..

[176] Ashraf G.M., Baeesa S.S. (2018). Investigation of Gal-3 Expression Pattern in Serum and Cerebrospinal Fluid of Patients Suffering From Neurodegenerative Disorders. Front. Neurosci..

[177] Tao C.C., Cheng K.M., Ma Y.L., Hsu W.L., Chen Y.C., Fuh J.L., Lee W.J., Chao C.C., Lee E.H.Y. (2020). Galectin-3 promotes Abeta oligomerization and Abeta toxicity in a mouse model of Alzheimer’s disease. Cell Death Differ..

[178] Seki T., Kanagawa M., Kobayashi K., Kowa H., Yahata N., Maruyama K., Iwata N., Inoue H., Toda T. (2020). Galectin 3-binding protein suppresses amyloid-beta production by modulating beta-cleavage of amyloid precursor protein. J. Biol. Chem..

[179] Anacker C., Hen R. (2017). Adult hippocampal neurogenesis and cognitive flexibility—Linking memory and mood. Nat. Rev. Neurosci..

[180] Miller S.M., Sahay A. (2019). Functions of adult-born neurons in hippocampal memory interference and indexing. Nat. Neurosci..

[181] Chen Y.C., Ma Y.L., Lin C.H., Cheng S.J., Hsu W.L., Lee E.H. (2017). Galectin-3 Negatively Regulates Hippocampus-Dependent Memory Formation through Inhibition of Integrin Signaling and Galectin-3 Phosphorylation. Front. Mol. Neurosci..

[182] Fernandez A.M., Torres-Aleman I. (2012). The many faces of insulin-like peptide signalling in the brain. Nat. Rev. Neurosci..

[183] Ziegler A.N., Levison S.W., Wood T.L. (2015). Insulin and IGF receptor signalling in neural-stem-cell homeostasis. Nat. Rev. Endocrinol..

[184] Bonds J.A., Shetti A., Stephen T.K.L., Bonini M.G., Minshall R.D., Lazarov O. (2020). Deficits in hippocampal neurogenesis in obesity-dependent and -independent type-2 diabetes mellitus mouse models. Sci. Rep..

[185] Karlsen A.E., Storling Z.M., Sparre T., Larsen M.R., Mahmood A., Storling J., Roepstorff P., Wrzesinski K., Larsen P.M., Fey S. (2006). Immune-mediated beta-cell destruction in vitro and in vivo-A pivotal role for galectin-3. Biochem. Biophys. Res. Commun..

[186] Darrow A.L., Shohet R.V., Maresh J.G. (2011). Transcriptional analysis of the endothelial response to diabetes reveals a role for galectin-3. Physiol. Genom..

[187] Barbagallo M., Dominguez L.J. (2014). Type 2 diabetes mellitus and Alzheimer’s disease. World J. Diabetes.

[188] Mehina E.M.F., Taylor S., Boghozian R., White E., Choi S.E., Cheema M.S., Korbelin J., Brown C.E. (2021). Invasion of phagocytic Galectin 3 expressing macrophages in the diabetic brain disrupts vascular repair. Sci. Adv..

[189] Machala E.A., McSharry B.P., Rouse B.T., Abendroth A., Slobedman B. (2019). Gal power: The diverse roles of galectins in regulating viral infections. J. Gen. Virol..

[190] Wang W.H., Lin C.Y., Chang M.R., Urbina A.N., Assavalapsakul W., Thitithanyanont A., Chen Y.H., Liu F.T., Wang S.F. (2019). The role of galectins in virus infection—A systemic literature review. J. Microbiol. Immunol. Infect..

[191] Vasta G.R. (2009). Roles of galectins in infection. Nat. Rev. Microbiol..

[192] Shan C., Xia H., Haller S.L., Azar S.R., Liu Y., Liu J., Muruato A.E., Chen R., Rossi S.L., Wakamiya M. (2020). A Zika virus envelope mutation preceding the 2015 epidemic enhances virulence and fitness for transmission. Proc. Natl. Acad. Sci. USA.

[193] Yang M.L., Chen Y.H., Wang S.W., Huang Y.J., Leu C.H., Yeh N.C., Chu C.Y., Lin C.C., Shieh G.S., Chen Y.L. (2011). Galectin-1 binds to influenza virus and ameliorates influenza virus pathogenesis. J. Virol..

[194] Lenza M.P., Oyenarte I., Diercks T., Quintana J.I., Gimeno A., Coelho H., Diniz A., Peccati F., Delgado S., Bosch A. (2020). Structural Characterization of N-Linked Glycans in the Receptor Binding Domain of the SARS-CoV-2 Spike Protein and their Interactions with Human Lectins. Angew. Chem. Int. Ed. Engl..

[195] Casalino L., Gaieb Z., Goldsmith J.A., Hjorth C.K., Dommer A.C., Harbison A.M., Fogarty C.A., Barros E.P., Taylor B.C., McLellan J.S. (2020). Beyond Shielding: The Roles of Glycans in the SARS-CoV-2 Spike Protein. ACS Cent. Sci..

[196] Shajahan A., Supekar N.T., Gleinich A.S., Azadi P. (2020). Deducing the N- and O-glycosylation profile of the spike protein of novel coronavirus SARS-CoV-2. Glycobiology.

[197] Shang J., Ye G., Shi K., Wan Y., Luo C., Aihara H., Geng Q., Auerbach A., Li F. (2020). Structural basis of receptor recognition by SARS-CoV-2. Nature.

[198] Gutmann C., Takov K., Burnap S.A., Singh B., Ali H., Theofilatos K., Reed E., Hasman M., Nabeebaccus A., Fish M. (2021). SARS-CoV-2 RNAemia and proteomic trajectories inform prognostication in COVID-19 patients admitted to intensive care. Nat. Commun..

[199] McCullough P.A., Olobatoke A., Vanhecke T.E. (2011). Galectin-3: A novel blood test for the evaluation and management of patients with heart failure. Rev. Cardiovasc. Med..

[200] Newlaczyl A.U., Yu L.G. (2011). Galectin-3—a jack-of-all-trades in cancer. Cancer Lett.

[201] Oberg C.T., Leffler H., Nilsson U.J. (2011). Inhibition of galectins with small molecules. Chimia.

[202] Strik H.M., Kolodziej M., Oertel W., Basecke J. (2011). Glycobiology in Malignant Gliomas: Expression and Functions of Galectins and Possible Therapeutic Options. Curr. Pharm. Biotechnol..

[203] McCullough P.A. (2014). Practical experience using galectin-3 in heart failure. Clin. Chem. Lab. Med..

[204] Caniglia J.L., Guda M.R., Asuthkar S., Tsung A.J., Velpula K.K. (2020). A potential role for Galectin-3 inhibitors in the treatment of COVID-19. PeerJ.

[205] Uchino Y., Woodward A.M., Mauris J., Peterson K., Verma P., Nilsson U.J., Rajaiya J., Argueso P. (2018). Galectin-3 is an amplifier of the interleukin-1beta-mediated inflammatory response in corneal keratinocytes. Immunology.

[206] Cerliani J.P., Stowell S.R., Mascanfroni I.D., Arthur C.M., Cummings R.D., Rabinovich G.A. (2011). Expanding the Universe of Cytokines and Pattern Recognition Receptors: Galectins and Glycans in Innate Immunity. J. Clin. Immunol..

[207] Rabinovich G.A., Toscano M.A., Jackson S.S., Vasta G.R. (2007). Functions of cell surface galectin-glycoprotein lattices. Curr. Opin. Struct. Biol..

[208] Du Toit A. (2014). Endocytosis: Bend it like galectin 3. Nat. Rev. Mol. Cell Biol..

[209] Li F. (2016). Structure, Function, and Evolution of Coronavirus Spike Proteins. Annu. Rev. Virol..

[210] Peng G., Sun D., Rajashankar K.R., Qian Z., Holmes K.V., Li F. (2011). Crystal structure of mouse coronavirus receptor-binding domain complexed with its murine receptor. Proc. Natl. Acad. Sci. USA.

[211] Paterson R.W., Brown R.L., Benjamin L., Nortley R., Wiethoff S., Bharucha T., Jayaseelan D.L., Kumar G., Raftopoulos R.E., Zambreanu L. (2020). The emerging spectrum of COVID-19 neurology: Clinical, radiological and laboratory findings. Brain.

[212] Sun X.L. (2020). The Role of Cell Surface Sialic Acids for SARS-CoV-2 Infection. Glycobiology.

[213] Szele F.G., Chesselet M.F. (1996). Cortical lesions induce an increase in cell number and PSA-NCAM expression in the subventricular zone of adult rats. J. Comp. Neurol..

[214] Ramirez Hernandez E., Sanchez-Maldonado C., Mayoral Chavez M.A., Hernandez-Zimbron L.F., Patricio Martinez A., Zenteno E., Limon Perez de Leon I.D. (2020). The therapeutic potential of galectin-1 and galectin-3 in the treatment of neurodegenerative diseases. Expert Rev. Neurother..

[215] Sakaguchi M., Shingo T., Shimazaki T., Okano H.J., Shiwa M., Ishibashi S., Oguro H., Ninomiya M., Kadoya T., Horie H. (2006). A carbohydrate-binding protein, Galectin-1, promotes proliferation of adult neural stem cells. Proc. Natl. Acad. Sci. USA.

[216] Whitney P.L., Powell J.T., Sanford G.L. (1986). Oxidation and chemical modification of lung beta-galactoside-specific lectin. Biochem. J..

[217] Morshead C.M., Reynolds B.A., Craig C.G., McBurney M.W., Staines W.A., Morassutti D., Weiss S., van der Kooy D. (1994). Neural stem cells in the adult mammalian forebrain: A relatively quiescent subpopulation of subependymal cells. Neuron.

[218] Imaizumi Y., Sakaguchi M., Morishita T., Ito M., Poirier F., Sawamoto K., Okano H. (2011). Galectin-1 is expressed in early-type neural progenitor cells and down-regulates neurogenesis in the adult hippocampus. Mol. Brain.

[219] Sakaguchi M., Imaizumi Y., Shingo T., Tada H., Hayama K., Yamada O., Morishita T., Kadoya T., Uchiyama N., Shimazaki T. (2010). Regulation of adult neural progenitor cells by Galectin-1/beta1 Integrin interaction. J. Neurochem..

[220] Ishibashi S., Kuroiwa T., Sakaguchi M., Sun L., Kadoya T., Okano H., Mizusawa H. (2007). Galectin-1 regulates neurogenesis in the subventricular zone and promotes functional recovery after stroke. Exp. Neurol..

[221] Han H., Xia Y., Wang S., Zhao B., Sun Z., Yuan L. (2011). Synergistic effects of galectin-1 and reactive astrocytes on functional recovery after contusive spinal cord injury. Arch. Orthop. Trauma Surg..

[222] Yamane J., Nakamura M., Iwanami A., Sakaguchi M., Katoh H., Yamada M., Momoshima S., Miyao S., Ishii K., Tamaoki N. (2010). Transplantation of galectin-1-expressing human neural stem cells into the injured spinal cord of adult common marmosets. J. Neurosci. Res..

[223] Kajitani K., Nomaru H., Ifuku M., Yutsudo N., Dan Y., Miura T., Tsuchimoto D., Sakumi K., Kadoya T., Horie H. (2009). Galectin-1 promotes basal and kainate-induced proliferation of neural progenitors in the dentate gyrus of adult mouse hippocampus. Cell Death Differ..

[224] Sakaguchi M., Arruda-Carvalho M., Kang N.H., Imaizumi Y., Poirier F., Okano H., Frankland P.W. (2011). Impaired spatial and contextual memory formation in galectin-1 deficient mice. Mol. Brain.

[225] Akers K.G., Martinez-Canabal A., Restivo L., Yiu A.P., De Cristofaro A., Hsiang H.L., Wheeler A.L., Guskjolen A., Niibori Y., Shoji H. (2014). Hippocampal neurogenesis regulates forgetting during adulthood and infancy. Science.

