# Supplementary material for: Novel Galectin-3 Roles in Neurogenesis, Inflammation and Neurological Diseases
Source: Cells. 2021 Nov 5;10(11):3047. doi: 10.3390/cells10113047 (PMC8618878; doi:10.3390/cells10113047)
Supplement: Supplementary file 1 [file cells-10-03047-s001.zip › cells-1345929-supplementary.pdf]

## Supplementary material

## Methods for Figure 2.

Ethics

All mouse procedures were carried out in accordance to Home Office UK regulations and the Animals (Scientific Procedures) Act 1986. All mice were housed at the animal unit at Functional Genomics Facility, Wellcome Trust Centre for Human Genetics, Oxford University.

Brain sections

Brain sections of TamIDH1-KI mice were obtained from Drs. Chiara Bardella and and Ian Tomlinson, Wellcome Trust Centre for Human Genetics, University of Oxford. To obtain animal lineages with $IDH1^{R132H}$ mutations at the SVZ, $Idh1fl^{(R132H)/+}$ mice were crossed with mice carrying a tamoxifen-inducible nestin-CreER(T2), resulting in Nes-CreER(T2); $Idh1fl^{(R132H)/+}$mice. Those mice were then treated with tamoxifen at 4–6 weeks of age for 5 consecutive days (TamIDH1-KI) (Bardella et al 2016).

Immunofluorescence of free-floating brain sections

For immunofluorescence (IF), free-floating SVZ sections (up to 6/animal) were placed in plates of 12-wells containing phosphate buffered saline [PBS – 8 g Sodium chloride (NaCl) + 240 mg Potassium dihydrogen phosphate (KH2PO4) + 1.44 g Sodium phosphate dibasic anhydrous (Na2HPO4) in distilled water, pH 7.4] for an initial wash of the cryoprotectant. All the washes were performed on a rotating shaker. Porous well inserts (Sigma-Aldrich) were used to facilitate transference of the sections between wells. In order to avoid possible background caused by the fixation process, a step of 15 min incubation with 50 mM glycine in PBS was conducted, followed by another round of PBS washes (3 times of 10 min each). Nonspecific sites of antibody binding were blocked with PBS+ solution (PBS + 10% Donkey serum + 0.1% Triton-X-100) for 1 h at room temperature (RT). Primary antibody incubation was conducted overnight at 4°C with antibodies diluted in PBS+ at different concentrations (Table S1). The next day, the excess antibody was washed away with a set of PBS washes (3 times 10 min each), which preceded 1 h RT incubation with secondary antibodies diluted at 1:500 in PBS+. Secondary antibodies were species-specific and conjugated with Alexa Fluor 488, 568, 594, or 647 fluorophores (Invitrogen and Sigma-Aldrich). This step and the following ones were conducted in darkness to protect fluorophores from bleaching. After washes with PBS (3 times of 10 min each), the nuclei were stained with 4',6-diamidino-2-phenylindole (DAPI, Sigma-Aldrich). Cells were then incubated for 15 min with Alexa Fluor 568 Phalloidin (ThermoFisher Scientific) diluted at 1:20 in PBS. For DAPI staining, cells were incubated for 10 min RT with 10 µg/ml DAPI diluted in PB. A final set of 3 times 10 min wash with PB was conducted before sections were mounted on Superfrost PlusTM slides (Thermo Fisher Scientific). After air-drying for approximately 30 min, slides were mounted with FluorSave mounting media (Calbiochem) and stored at 4°C in darkness.

Image analysis and quantifications of brain sections

A ZEISS LSM 710 Laser Scanning Confocal Microscope was used to take images of at least 3 SVZ sections per brain (technical replicates). Z-stacks with 1.0 µm interval were acquired using a 20X (Zeiss Plan-Neofluar 20X/0.50) or 40X (Zeiss Plan-NeoFluar 40X1.3 Oil DIC) objective lens. To allow quantification, a minimum of 3 images of the SVZ and adjacent striatum were acquired per section, which were analysed using the FIJI (NIH ImageJ) software package.
The activation state of at least 100 microglial cells was calculated as a percentage of microglia classified as activated, intermediate or resting against total Iba1+ cells per region.
Non-specific binding was always assessed with a secondary antibody-only negative control for immunofluorescence staining. Only experiments that lacked non- specific binding were used.

Cell culture

The glioblastoma multiforme (GBM) cell lines were kindly provided by Dr. Val Millar (Oxford Target Discovery Institute). Cells were maintained Dulbecco's Modified Eagle Medium (DMEM) supplemented with 10% Fetal Bovine Serum (FBS), 2 mM Glutamine, and Penicillin/Streptomycin (PenStrept) (100 U/ml) and cultured in a humid incubator at 37°C and 5% CO2.

Immunofluorescence of GBM cell lines

For immunofluorescence of GBM cells, 13 mm circular coverslips were added to the culture plates. Cover slips with attached cells were collected from the plate and fixed with PFA 4% (Thermo Fisher Scientific) for 15 min at RT in darkness. After fixation, coverslips were kept at 4°C in PBS or immediately permeabilised for 15 min with 0.5% TritonTM X-100 (Sigma-Aldrich) in PBS. Coverslips were blocked at RT for 1 h with blocking solution (5% BSA + 0.2% TritonTM X-100 in PBS) and then incubated with primary antibodies at RT for 2 h. Primary antibodies were diluted in antibody solution (1% BSA + 0.2% TritonTM X-100 in PBS) at different concentrations (Table S1) and a volume of 50 µl was enough to completely cover the area of the coverslips. After washing by an approximately 30 sec immersion in PBS, cover slips were incubated for 1 h at RT with secondary antibodies diluted in antibody solution at 1:1000 plus 10 µg/ml DAPI (Thermo Fisher Scientific). Secondary antibodies were species-specific and conjugated with Alexa Fluor 488, 568, or 647 fluorophores (Invitrogen). Following the incubation with secondary antibodies, coverslips were washed with PBS as described. Coverslips were mounted using ProLongTM Gold Antifade Mountant (Invitrogen), sealed with nail polish and air-dried overnight at 4°C in darkness. Images were acquired using Nikon Eclipse Ni-E Microscope or ZEISS LSM 780 Laser Scanning Microscope and processed in FIJI (ImageJ) software. Non-specific binding was always assessed with a secondary antibody-only negative control for immunofluorescence staining. Only experiments that lacked non-specific binding were used.

Table S1: Antibodies used for immunofluorescence

| **Antigen** | **Host** | **Clonality** | **Dilution** | **Supplier** | **Catalogue** |
|---|---|---|---|---|---|
| Galectin-3 | Rat | Monoclonal | 1:100 | Santa Cruz | sc-23938 |
| Iba1 | Goat | Monoclonal | 1:300 | Abcam | ab5076 |

Statistics

All experiments were relicated at least three times. Analyses and graphs were acquired using GraphPad Prism 8 software and data is presented as mean ± standard error of the mean (SEM). Student’s t-test was used to assess difference between two groups. Significant difference is presented as * for $p < 0.05$, ** for $p < 0.01$, and *** for $p < 0.001$.
